# Characterization of Self-reported Improvements in Knowledge and Health Among Users of Flo Period Tracking App: Cross-sectional Survey

**DOI:** 10.2196/40427

**Published:** 2023-04-26

**Authors:** Liudmila Zhaunova, Ryan Bamford, Tara Radovic, Aidan Wickham, Kimberly Peven, Jazz Croft, Anna Klepchukova, Sonia Ponzo

**Affiliations:** 1 Flo Health LTU Vilnius Lithuania; 2 Flo Health UK Limited London United Kingdom; 3 Department of Psychology and Ergonomics Technische Universitaet Berlin Berlin Germany; 4 Maternal, Adolescent, Reproductive & Child Health Centre, London School of Hygiene & Tropical Medicine London United Kingdom; 5 Institute of Health Informatics University College London London United Kingdom

**Keywords:** health knowledge, menstrual cycle, pregnancy, period-tracking app, digital health, women’s health

## Abstract

**Background:**

Research shows that poor knowledge and awareness of menstrual and pregnancy health among women are associated with adverse reproductive health and pregnancy outcomes. Menstrual cycle– and pregnancy-tracking mobile apps are promising tools for improving women’s awareness of and attitudes toward their reproductive health; however, there is little information about subscribers’ perceptions of app functionality and its impact on their knowledge and health.

**Objective:**

This study aimed to explore knowledge and health improvements related to menstrual cycle and pregnancy, as well as improvements in general health among Flo app users. We also investigated what components of the Flo app were associated with the abovementioned improvements and evaluated whether those improvements differed based on education level, country of residence (low- and middle-income vs high-income countries), free or premium subscription to the app, short- or long-term use of the app, and frequency of use.

**Methods:**

Flo subscribers who had been using the app for no less than 30 days, completed a web-based survey. A total of 2212 complete survey responses were collected. The survey included demographic questions and questions about motivations guiding the use of the Flo app and which components of the app improved their knowledge and health, as well as to what extent.

**Results:**

Most study participants reported improvements in menstrual cycle (1292/1452, 88.98%) and pregnancy (698/824, 84.7%) knowledge from Flo app use. Participants with higher levels of education and those from high-income countries reported using the app predominantly for getting pregnant (*χ*^2^_1_=4.2, *P*=.04; *χ*^2^_1_=52.3, *P*<.001, respectively) and pregnancy tracking (*χ*^2^_1_=19.3, *P*<.001; *χ*^2^_1_=20.9, *P*=.001, respectively). Participants with less education reported using the app to avoid pregnancy (*χ*^2^_1_=4.2; *P*=.04) and to learn more about their body (*χ*^2^_1_=10.8; *P*=.001) and sexual health (*χ*^2^_1_=6.3; *P*=.01), while participants from low- and middle-income countries intended to mainly learn more about their sexual health (*χ*^2^_1_=18.2; *P*<.001). Importantly, the intended use of the app across education levels and country income levels matched areas in which they had gained knowledge and achieved their health goals upon use of the Flo app. Period, fertile days, and ovulation predictions as well as symptom tracking were consistently the top 3 components in the app that helped users with their cycle knowledge and general health. Reading articles or watching videos helped with users' education regarding their pregnancy. Finally, the strongest improvements in knowledge and health were observed in premium, frequent, and long-term users.

**Conclusions:**

This study suggests that menstrual health apps, such as Flo, could present revolutionary tools to promote consumer health education and empowerment on a global scale.

## Introduction

### Background

Women’s health has long been understudied and underfunded, compromising the amount of health information available to women as well as the quality of health care they receive [1]. Recently, more efforts have been made to include women in clinical trials, and more policies have been introduced to enhance and promote women’s health globally [2-4]. However, many cultures still hold different myths and taboos regarding the reproductive health and menstrual cycle, specifically. Studies have shown that there is a large knowledge gap among adolescent girls and adult women, especially in low- and middle-income countries (LMICs), about menstrual cycle health [5,6], fertility [5,7], healthy pregnancy [8,9], and overall women’s health and well-being [10,11]. For example, a North Indian study found that only 31% of respondents reported the right definition of the menstrual cycle [6], whereas an extremely high prevalence of inadequate knowledge of symptoms, risk factors, and complications of preeclampsia (88.4%) was found among pregnant women in Ghana [9].

In high-income countries (HICs), despite an increase in growing social and governmental momentum to improve reproductive health education [12-16], there is still a knowledge gap and misconceptions about reproductive and fertility health as well as reluctance to seek medical treatment for some health issues [17,18]. A Chicago-based study reported that one of the main reasons for a delayed fibroid diagnosis was the belief among women that heavy menstrual bleeding was normal [17]. Another study conducted on American women of reproductive age found that almost 60% of all surveyed women did not know when they had higher chances of conception during the menstrual cycle, while more than two-thirds were unaware that pain during menstruation (eg, owing to conditions such as endometriosis) may correlate with a woman’s ability to get pregnant [18]. A study conducted in England found that 30% of adolescent girls were unaware if their periods were regular; furthermore, most of them were considerably less aware of endometriosis than of other chronic diseases such as diabetes or epilepsy [19]. Another study found that only 42% of pregnant women in Italy knew of all the main risk factors in pregnancy such as alcohol consumption, smoking, passive smoking, and obesity [20]. Thus, low menstrual cycle and pregnancy health awareness among women and people who menstruate might cause adverse effects on women’s and newborn health and well-being and can restrict women from their daily sociocultural activities and impact their quality of life [21,22]. In addition, poor knowledge and misconceptions about reproductive health might delay the time to diagnosis and increase medical costs associated with treatment [17,23].

Given the lack of reproductive health knowledge among women, identifying medically credible, easy-to-access and understand sources of reproductive health–related information is crucial. On the basis of survey data, women’s health care providers (HCPs) are one of the main sources of trustworthy reproductive health–related knowledge; however, more than one-third of survey respondents reported visiting their reproductive HCP less than once per year or having never visited one [18]. Furthermore, the use of medical jargon or more dense language by HCPs can confuse patients and result in inability to recall the information that they received during medical consultations [24-27]. Health-related internet websites are another source of education and empowerment for women [18,28,29]. Nevertheless, web-based health sources vary in quality, accuracy, and reliability, and incorrect information or advice from websites may affect health-related behavior and decisions [30].

In the past few years, the rapid growth and acceptance of mobile health apps specifically designed to address women’s health needs have made substantial progress in the area of reproductive and pregnancy knowledge improvements [31-33]. Female health apps are often perceived as helpful by their users, as they provide easy-to-access information that helps them feel more knowledgeable and supported [34,35]. In addition to the educational component, apps for women’s health often provide their users with tracking functionality for their self-knowledge, monitoring, and recording [35,36]. Such functionality includes menstrual cycle and pregnancy tracking or tracking of cycle-related symptoms, for example, hormone-triggered migraines or mood swings [33,37-39]. Previous studies have shown that health mobile apps also facilitate good habit formation and health promotion by providing a number of functions to map behavior patterns across time, including, but not limited to, sleep, diet, physical activity, and mental health practices etc [40,41]. Despite the prevalence and apparent popularity of women health–tracking apps, there remains a knowledge gap regarding the accuracy and safety of the medical advice provided as well as the effectiveness of digital health products in changing health behaviors. Researchers, clinicians, and patient groups have recently advocated for more rigorous requirements regarding the validation and evaluation of digital health solutions, with scientists highlighting the need for a unified evidence generation framework [42-45]. As such, there is a need for health app developers to conduct and publish research assessing the effectiveness of products at both pre- and postmarket time points, so that end users and clinicians are provided with sufficient evidence to make an informed decision [46,47].

One of the mobile apps dedicated to women’s health and well-being is the Flo app (by Flo Health Inc). Flo offers its users artificial intelligence–based period and ovulation predictions and allows them to track their periods, fertile window, ovulation, and symptoms while trying to get pregnant or avoid pregnancy, as well as to track pregnancy symptoms and fetal development. In addition to the tracking functionality, Flo provides its users with evidence-based and expert-reviewed educational content available through the in-app library. Flo app content creation and validation are based on a peer-reviewed practice in which Flo’s in-house medical doctors and external medical and science experts review each piece of content before it is published in the Flo application or on the website. Each content unit (article, graphics, courses, etc) within the app has references to peer-reviewed articles, medical guidelines, and links to internationally recognized health advocacy organizations and academic institutions. Moreover, each article has a link to the profile of the medical expert or organization who reviewed it. To check for symptoms against an array of conditions, Flo also has interactive questions available via so-called “*health assistants*” or “*chatbots*” [38]. Finally, Flo has a secure place called “Secret Chats” where women can discuss intimate topics, ask questions anonymously, and obtain support from millions of women worldwide, thus reducing perceived taboos and stigma surrounding topics such as menstrual health and sexual life [48-50].

### Objective

Although the Flo app provides a range of functionalities, there is an open question as to whether the Flo users obtain any improvements in knowledge and health by using the app. Hence, the aim of this study was to describe the demographic and app use characteristics of a self-enrolled sample of Flo subscribers and explore the relationship between app use and self-reported improvements in knowledge and health related to the menstrual cycle and pregnancy, as well as improvements in general health in Flo users. We hypothesized that subscribers who use Flo Premium instead of a free version, as well as those who used the app more frequently and for a longer period, would be more likely to report increased health benefits with a greater improvement in their knowledge and understanding across different areas. We also aimed to evaluate the components of the Flo app that were associated with the aforementioned improvements.

## Methods

### Participants

Users of the Flo app who had the app installed in English for at least 30 days and were >18 years old were eligible to participate in this study. Recruitment took place within the Flo app between December 5, 2021, and January 16, 2022. We collected 5015 partial responses and 2212 complete survey responses. Only responses from the participants who fully completed the survey were used for the analysis (Table 1 provides the sample’s demographics).

**Table 1:** Demographics of study participants (N=2212).

Category			Frequency, n (%)
**Country (top 10)**			
	The United States	758 (34.3)	
	The United Kingdom	217 (9.8)	
	Canada	121 (5.5)	
	India	108 (4.9)	
	Australia	92 (4.2)	
	South Africa	86 (3.9)	
	Nigeria	82 (3.7)	
	Ghana	63 (2.8)	
	Philippines	43 (1.9)	
	Netherlands	32 (1.4)	
**Age (years)**			
	18-24	544 (24.6)	
	25-34	1264 (57.1)	
	35-44	370 (16.7)	
	45-54	34 (1.5)	
**Race**			
	White, European-American, or Caucasian	1041 (47)	
	Black or African American	397 (17.9)	
	Asian or Asian-American	223 (10.1)	
	Hispanic, Latino, Spanish origin	116 (5.2)	
	Biracial or multiracial	88 (4)	
	American Indian or Alaskan	12 (0.5)	
	Native Hawaiian or Pacific Islander	3 (0.1)	
	Other	225 (10.2)	
	Prefer not to say	107 (4.8)	
**Gender**			
	Woman	2181 (96.7)	
	Nonbinary	22 (1)	
	Genderqueer or gender fluid	19 (0.8)	
	Questioning or unsure	10 (0.4)	
	Man	5 (0.2)	
	Trans man	3 (0.1)	
	Trans woman	1 (0.1)	
	Agender	3 (0.1)	
	I prefer not to say	11 (0.5)	
**Education**			
	Doctorate degree	43 (1.9)	
	Master’s degree	373 (16.9)	
	Bachelor’s degree	759 (34.3)	
	Associate’s degree	139 (6.3)	
	High school graduate or diploma	600 (27.1)	
	Some high school, no diploma	115 (5.2)	
	I prefer not to say	112 (5.1)	
	Other	71 (3.2)	

### Materials

Participants completed a demographic questionnaire followed by an investigator-developed quantitative survey (Multimedia Appendix 1 provides the full list of survey questions). The survey included questions addressing the aim, frequency, and duration of the app use, as well as whether and what components of the Flo app (and to what extent) helped users to improve their knowledge and health. Specifically, the survey included questions addressing improvements in knowledge and health across 5 different areas: menstrual health and education, irregular cycle and related conditions, getting pregnant and pregnancy health, general health (mental, sexual, physical health, and health behaviors), and communication with an HCP.

Overall, the survey contained 70 questions. Not every question was available for every participant, as questions were presented only if they were relevant to the participant based on their previous responses. For example, those who reported that they downloaded the app to track their pregnancy were not asked menstrual cycle–related questions. The survey was created using SurveyMonkey software (Momentive Inc).

### Procedure

Participants were notified of the possibility of participating in a survey via an in-app notification. Upon clicking on the survey button, the participants were redirected to SurveyMonkey and asked to provide electronic informed consent. Those who consented proceeded to complete the survey on their electronic devices, which took an average of 8 minutes.

### Ethical Considerations

The study was reviewed by the Independent Ethical Review Board (WIRB-Copernicus Group Institutional Review Board), which deemed the study exempt (IRB tracking number: 20216374 ).

### Defining Categories

To describe app use patterns among Flo users, the following categories were defined: a short-term user was any participant who had been using the Flo app for <1 year, whereas a long-term user was anyone using the Flo app for ≥1 year. A frequent user was defined as a participant who used the app several times a day to several times a week, whereas an infrequent user was a participant who used the app once a week or less. Premium app users are those who use the paid version of the Flo app, while free app users are those who use the free version of the app. Compared with the free version, the paid app version provides users with full access to all in-app content materials (including text and video materials), all symptom- and cycle-related chats with Flo’s health assistant, and all community discussion chats with an additional opportunity to ask for support and advice from a medical professional (“Ask an Expert”). Multimedia Appendix 2 lists the complete app use characteristics.

Users with higher education were defined as those who had either a bachelor’s, master’s, or doctorate degree, whereas users with less education included those with any education below a completed bachelor’s degree. In terms of age, a younger user was anyone between the ages of 18 and 34 years, whereas older users were defined as anyone between the ages of 35 and 54 years (Table 1).

Country income level was defined based on the World Bank Country Income classification [51]. In this study, 62.4% (78/125) of countries were defined as LMICs, whereas the remaining 37.6% (47/125) of countries were classified as HICs. Multimedia Appendices 3 and 4 provide a complete breakdown of HICs and LMICs, respectively.

### Statistical Analysis

Data were analyzed using Python Jupyter Notebook (version 6.0.1; Project Jupyter). All variables in the survey were categorical. Throughout the survey, participants had options to answer questions as binary categorical variables (yes or no), nominal categorical variables (eg, symptoms they believe Flo has helped with) and ordinal categorical variables (eg, 5 categories from “Not at all” to “A great deal”). Throughout the survey, depending on the participants’ choices, the users were directed to answer different questions. Consequently, the number of participants who answered each question differed. Ordinal categorical variables were recoded as dichotomous variables (not at all—a little vs a moderate amount—a great deal).

Chi-squared tests compared participant responses for the association between user app subscription status (free vs premium), frequency of app use (frequent vs infrequent), and duration of use (short term vs long term). Chi-squared tests were also used to assess whether the country of residence and education level had a relationship with the reasons why they used the app, the areas in which users’ knowledge was improved, and to what extent knowledge was improved. To confirm the relationship between demographic factors (age and education) and the reasons for using the app, we fitted univariate logistic regression models.

## Results

### Overall Knowledge and Health Improvements Upon Flo App Use

Most respondents reported that the Flo app had improved how educated they feel about their overall cycle (1292/1452, 88.98%; Table S1 in Multimedia Appendix 5, question 46) and pregnancy health (698/824, 84.7%; Table S2 in Multimedia Appendix 5, question 34). Users who had tracked their cycle with Flo felt improvements in the following top 3 menstrual health areas: they knew whether their cycle was regular (1085/1292, 84.0%), whether their cycle length was normal (978/1292, 75.7%), and whether it was normal to have certain symptoms during their cycle (755/1292, 58.4%; Table S1 in Multimedia Appendix 5, question 47). Users also reported that they believed Flo had helped them understand how to use their cycle to know when they were most fertile (780/1292, 60.4%) and how their cycle affected their physical (776/1292, 60.1%) and mental health (765/1292, 59.2%; Table S1 in Multimedia Appendix 5, question 51). In addition, 77.3% (1270/1642) of participants reported that Flo had helped them manage their menstrual symptoms, with the top 3 symptoms being “ovulation” (668/1270, 52.6%); “bad mood” (including feeling sad, guilty, irritated, obsessive thoughts, and confused or self-critical; 641/1270, 50.5%); and “cramps” (618/1270, 48.7%; Table S1 in Multimedia Appendix 5; questions 16 and 17). Finally, 65.1% (309/475) of users said that the app had helped them manage their irregular cycles and related conditions (Table S1 in Multimedia Appendix 5, question 21).

Most of the study cohort (627/824, 76.1%) who used the app to track their pregnancy reported getting pregnant while using Flo (Table S2 in Multimedia Appendix 5, question 29). Of those who became pregnant, 73.5% (461/627) of believed that using Flo had helped them become pregnant (Table S2 in Multimedia Appendix 5, question 30). More than 8 in 10 participants (530/627, 84.5%) said that Flo had helped them to prepare for a healthy pregnancy (Table S2 in Multimedia Appendix 5, question 32). Pregnancy-tracking users reported that Flo had improved their knowledge about their body during pregnancy (589/698, 84.4%); their baby’s development (577/698, 82.7%); and which symptoms did not require medical attention and which did during pregnancy (505/698, 72.3%; Table S2 in Multimedia Appendix 5, question 36). The study participants also reported that Flo had improved their understanding of how pregnancy affected their physical health (509/698, 72.9%); how to manage their pregnancy symptoms (403/698, 57.7%); and how to optimize their life around their pregnancy (393/698, 56.3%; Table S2 in Multimedia Appendix 5, question 39).

A smaller number of respondents reported improvements in mental (843/2212, 38.1%), sexual (835/2212, 37.7%), and physical health (589/2212, 26.6%); health behaviors (621/2212, 28.1%); and communication with an HCP (587/2212, 26.5%; Table S3 in Multimedia Appendix 5, question 53).

Period predictions, fertile days and ovulation predictions, and symptom tracking were the top 3 components of the app that have helped users with all the abovementioned areas (Tables S1 and S3 in Multimedia Appendix 5) except pregnancy education where “reading articles or watching video sources” in the app was rated the highest (503/698, 72.1%; Table S2 in Multimedia Appendix 5, question 38). Complete responses regarding knowledge and health improvements in cycle, pregnancy, and general health are shown in Tables S1, S2, and S3 in Multimedia Appendix 5, respectively.

### Knowledge and Health Improvements in Relation to Education Level

Study participants with less education were more likely to use the Flo app to track irregular cycles and related conditions to learn more about their body and sexual health and to help them not become pregnant (*χ*^2^_1_=10.9, *P*<.001; *χ*^2^_1_=10.8, *P*=.001; *χ*^2^_1_=6.3, *P*=.01; *χ*^2^_1_=4.2, *P*=.04, respectively), while higher-educated users were more likely to use the app to help them become pregnant and for pregnancy tracking (*χ*^2^_1_=4.2, *P*=.04; *χ*^2^_1_=19.3, *P*<.001, respectively; Table 2). Complete statistics on the reasons for using the app in relation to age are shown in Table S1 in Multimedia Appendix 6.

**Table 2:** Education versus reasons for using the app (N=2212).

Reasons for using the app	HE^a^, n (%)	LE^b^, n (%)	Chi-square (*df*)	*P* value
Menstrual cycle and symptom tracking	943 (80.3)	851 (82.1)	1.1 (1)	.01
Irregular cycles and related conditions	219 (18.6)	254 (24.5)	10.9 (1)	<.001
Pregnancy tracking	487 (41.4)	335 (32.3)	19.3 (1)	<.001
To help me get pregnant	657 (55.9)	537 (51.5)	4.2 (1)	.04
To help me not get pregnant	117 (10)	133 (12.8)	4.2 (1)	.04
Pregnancy loss	73 (6.2)	78 (7.5)	1.3 (1)	.26
Sexual health	305 (26)	320 (30.9)	6.3 (1)	.01
Get tailored health information	390 (33.2)	343 (33.1)	0.0(1)	.99
To learn more about my body	444 (38)	467 (45)	10.8 (1)	.001

^a^HE: higher education.

^b^LE: lower education.

Univariate logistic regression models confirmed the results of the chi-squared tests. Participants with a higher educational background, for example, master’s degree, had significantly higher odds (odds ratio 1.80, 95% CI 1.15-2.86) of using the app for pregnancy tracking and significantly lower odds (odds ratio 0.59, 95% CI 0.39-0.91) of using the app to learn more about their body compared with users with “Some High School, no Diploma.” Tables S2 and S3 in Multimedia Appendix 6 show full logistic regression results for education and age, respectively.

Furthermore, we examined whether both lower- and higher-educated users achieved health and knowledge improvements in the intended areas of app use. Users with less education were more likely to report that Flo had helped them improve their understanding of how to manage their menstrual symptoms (*χ*^2^_1_=4.7; *P*=.03; Table S1 in Multimedia Appendix 7, question 51) and helped them identify issues related to endometriosis (*χ*^2^_1_=8.0; *P*=.004; Table S1 in Multimedia Appendix 7, question 47). In addition, they were more likely to report that Flo had improved their sexual health knowledge (*χ*^2^_1_=9.5; *P*=.002), such as sexually transmitted infections (STIs) and how to avoid them (*χ*^2^_1_=13.1; *P*<.001); the signs and symptoms of STIs (*χ*^2^_1_=19.3; *P*<.001); safe sex (*χ*^2^_1_=11.7; *P*<.001); contraception options (*χ*^2^_1_=11.1; *P*<.001); the signs and symptoms during sex that were indicative of a health issue (*χ*^2^_1_=10.6; *P*<.001); and how to have more pleasure during sex (*χ*^2^_1_=6.7; *P*=.009; Table S2 in Multimedia Appendix 7, questions 53, 61, and 62).

Finally, users with less education were more likely to self-report that Flo had helped them improve their skin (*χ*^2^_1_=4.8; *P*=.03) or fitness (*χ*^2^_1_=5.4; *P*=.02) from a moderate to a great deal (Table S2 in Multimedia Appendix 7, question 57). In addition, they reported that Flo had helped them reduce harmful habits (*χ*^2^_1_=12.1; *P*<.001; Table S2 in Multimedia Appendix 7, question 59) and made them more confident about asking their HCPs for resources that they thought they needed (eg, stress relief, birth control, and support: *χ*^2^_1_=4.5; *P*=.03; Table S2 in Multimedia Appendix 7, question 64).

In contrast, users with higher education mainly reported improvements in pregnancy health education (Table S3 in Multimedia Appendix 7, questions 34, 36, and 39). In addition, they were more likely to report that they conceived while using Flo (*χ*^2^_1_=17.8; *P*<.001; Table S3 in Multimedia Appendix 7, question 29). Complete responses regarding knowledge and health improvements in cycle health, general health, and pregnancy health in relation to education level are shown in Tables S1, S2, and S3, respectively, in Multimedia Appendix 7.

### Knowledge and Health Improvements in Relation to Country of Residence (LMICs vs HICs)

The study included participants from both HICs (1513/2212, 68.4%) and LMICs (699/2212, 31.6%), with participants from LMICs having a statistically lower level of education compared with those from HICs (*χ*^2^_1_=11.0; *P*<.001). Table 3 shows that participants from HICs were more likely to use the app for menstrual cycle and symptom tracking, for pregnancy tracking, to get help with getting pregnant, and for pregnancy loss (*χ*^2^_1_=11.8, *P*<.001; *χ*^2^_1_=20.9, *P*<.001; *χ*^2^_1_=52.3, *P*<.001; *χ*^2^_1_=22.6, *P*<.001, respectively), while participants from LMICs were more likely to use the app to improve their sexual health (*χ*^2^_1_=18.2; *P*<.001).

**Table 3:** Low- and middle-income countries (LMICs) and high-income countries (HICs) versus reasons for using the app (N=2212).

Reasons for using the app	HIC, n (%)	LMIC, n (%)	Chi-square (*df*)	*P* value
Menstrual cycle and symptom tracking	1257 (83.1)	537 (76.9)	11.8 (1)	<.001
Irregular cycles and related conditions	324 (21.4)	149 (21.3)	0.0 (1)	.99
Pregnancy tracking	611 (40.4)	211 (30.2)	20.9 (1)	<.001
To help me get pregnant	894 (59.1)	297 (42.5)	52.3 (1)	<.001
To help me not get pregnant	172 (11.4)	78 (11.2)	0.0(1)	.94
Pregnancy loss	130 (8.6)	21 (3)	22.6 (1)	<.001
Sexual health	385 (25.4)	240 (34.4)	18.2 (1)	<.001
Get tailored health information	502 (33.2)	231 (33)	0.0(1)	.99
To learn more about my body	611 (40.4)	303 (43.4)	1.6 (1)	.20

We further tested whether users from LMICs and HICs achieved their health and knowledge improvement in their intended areas of app use. We found that users from HICs were significantly more likely to report that they became pregnant while using the Flo app (*χ*^2^_1_=17.0; *P*<.001; Table S1 in Multimedia Appendix 8, question 29), as well as know more about their baby’s development (*χ*^2^_1_=4.2; *P*=.04; Table S1 in Multimedia Appendix 8, question 36) and communicate better with their HCPs (*χ*^2^_1_=6.2; *P*=.01; Table S2 in Multimedia Appendix 8, question 53) and partners about their cycle (*χ*^2^_1_=5.7; *P*=.02; Table S3 in Multimedia Appendix 8, question 47).

Users from LMICs were significantly more likely to self-report that using Flo had helped them improve their understanding of sexual health (*χ*^2^_1_=17.7; *P*<.001; Table S2 in Multimedia Appendix 8, question 53)—more specifically, their understanding of STIs and how to avoid them (*χ*^2^_1_=8.1; *P*=.004; Table S2 in Multimedia Appendix 8, question 61) and how to have safe sex (*χ*^2^_1_=17.6; *P*<.001; Table S2 in Multimedia Appendix 8, question 61), as well as their understanding of their sexuality (*χ*^2^_1_=14.7; *P*<.001; Table S2 in Multimedia Appendix 8, question 62) and how to get more pleasure during sex (*χ*^2^_1_=5.0; *P*=.03; Table S2 in Multimedia Appendix 8, question 62). In addition to improving their sexual health, users from LMICs also reported improvements in understanding of whether their period flow was normal or not (*χ*^2^_1_=6.3; *P*=.01; Table S3 in Multimedia Appendix 8, question 47); how to identify issues related to polycystic ovary syndrome (PCOS; *χ*^2^_1_=6.7; *P*=.001; Table S3 in Multimedia Appendix 8, question 47); and how to manage menstrual symptoms (*χ*^2^_1_=23.8; *P*<.001; Table S3 in Multimedia Appendix 8, question 51) and postpartum symptoms (*χ*^2^_1_=8.9; *P*=.002; Table S1 in Multimedia Appendix 8, question 39). Finally, users from LMICs also reported improvements in physical (*χ*^2^_1_=25.1; *P*<.001; Table S2 in Multimedia Appendix 8, question 53) and mental health (*χ*^2^_1_=4.7; *P*=.03; Table S2 in Multimedia Appendix 8, question 53), including having more energy (*χ*^2^_1_=4.5; *P*=.03; Table S2 in Multimedia Appendix 8, question 57); sleeping better (*χ*^2^_1_=6.0; *P*=.01; Table S2 in Multimedia Appendix 8, question 57); having better stress management skills (*χ*^2^_1_=5.0; *P*=.03; Table S2 in Multimedia Appendix 8, question 59); and reducing harmful habits (*χ*^2^_1_=5.0, *P*=.03; Table S2 in Multimedia Appendix 8, question 59). Complete results regarding knowledge and health improvements in the pregnancy, general health, and cycle health between HICs and LMICs are shown in Tables S1, S2, and S3, respectively, in Multimedia Appendix 8.

### Knowledge and Health Improvements in Relation to Paid Versus Free App Use

Similar to users with higher education, Flo Premium users reported using the app mainly for pregnancy-related issues (pregnancy tracking: *χ*^2^_1_=109.8, *P*<.001; to help get pregnant: *χ*^2^_1_=21.4, *P*<.001) as well as to obtain tailored health information relevant to them (*χ*^2^_1_=16.7; *P*<.001; Table 4).

**Table 4:** Reasons for using the app versus app use characteristics (N=2212).

Reasons for using the app	F^a^, n (%)	P^b^, n (%)	F vs P			LT^c^, n (%)		ST^d^, n (%)		LT vs ST			Fr^e^, n (%)		I^f^, n (%)		Fr vs I	
			Chi-square (*df*)	*P* value					Chi-square (*df*)		*P* value					Chi-square (*df*)		*P* value
Menstrual cycle and symptom tracking	484 (84.9)	674 (80.8)	3.7 (1)	.06	719 (88.4)		439 (74.3)		46.5 (1)		<.001	889 (82.2)		267 (83.3)		0.1 (1)		.73
Irregular cycles and related conditions	132 (23.2)	170 (20.4)	1.4 (1)	.24	194 (23.9)		108 (18.3)		6.0 (1)		.01	217 (20.1)		85 (26.3)		5.4 (1)		.02
Pregnancy tracking	137 (24)	435 (52.2)	109.8 (1)	<.001	338 (41.7)		234 (39.6)		0.5 (1)		.49	481 (44.5)		91 (28.2)		26.8 (1)		<.001
To help me get pregnant	273 (47.9)	505 (60.6)	21.4 (1)	<.001	403 (49.6)		375 (63.5)		26.1 (1)		<.001	643 (59.5)		135 (41.8)		30.8 (1)		<.001
To help me not get pregnant	69 (12.1)	84 (10.1)	1.2 (1)	.27	121 (14.9)		32 (5.4)		30.6 (1)		<.001	114 (10.5)		39 (12.1)		0.5 (1)		.50
Pregnancy loss	23 (4)	75 (9)	12.1 (1)	<.001	61 (7.5)		37 (6.3)		0.6 (1)		.43	85 (7.9)		13 (4)		5.1 (1)		.02
Sexual health	175 (30.7)	234 (28.1)	1.0 (1)	.31	273 (33.6)		136 (23)		18.0 (1)		<.001	315 (29.1)		94 (29.1)		0.0 (1)		.99
Get tailored health information	163 (28.6)	328 (39.3)	16.7 (1)	<.001	310 (38.1)		181 (30.6)		8.1 (1)		.004	389 (36)		102 (31.6)		1.9 (1)		.16
Learn more about my body	233 (40.9)	351 (42.1)	0.2 (1)	.69	368 (45.3)		216 (36.5)		10.3 (1)		.001	456 (42.2)		128 (39.6)		0.6 (1)		.45

^a^P: premium app user.

^b^F: free app user.

^c^LT: long-term app user.

^d^ST: short-term app user.

^e^Fr: frequent user of the app.

^f^I: infrequent user of the app.

We investigated whether Flo Premium users achieved health and knowledge improvements in their intended areas of the app use. First, we found that premium users of Flo were more likely to report knowledge improvements in almost all areas of pregnancy health (Table S1 in Multimedia Appendix 9, questions 29, 32, 34, 36, and 39). In addition, premium users were more likely to report knowledge and health improvements regarding their menstrual health (Table S2 in Multimedia Appendix 9, questions 17, 46, 47, and 51). Finally, Flo Premium users were more likely to report that Flo had helped them improve all aspects of general health (except sexual health), as well as improve communication with HCPs; specifically, Flo Premium users felt more confident in sharing what was going on in their body when communicating with their HCPs (Table S3 in Multimedia Appendix 9, questions 53 and 64).

Complete responses regarding knowledge and health improvements in pregnancy, cycle, and general health in relation to paid versus free app use are shown in Tables S1, S2, and S3, respectively, in Multimedia Appendix 9.

### Knowledge and Health Improvements in Relation to Frequency of the App Use

Similar to users with higher education and Flo Premium users, participants who used the app frequently reported using the app mainly for pregnancy-related issues (pregnancy tracking: *χ*^2^_1_=26.8; *P*<.001; to get help with becoming pregnant: *χ*^2^_1_=30.8; *P*<.001), whereas infrequent app users were more likely to report using the app to track irregular cycles and related conditions (*χ*^2^_1_=5.4; *P*=.02; Table 4).

We examined whether frequent users of Flo achieved pregnancy health and knowledge improvements from app use. As shown in Table S1 in Multimedia Appendix 10, frequent Flo app users were more likely to report that Flo app had helped them become pregnant (*χ*^2^_1_=4.5; *P*=.03; question 30); prepare for a healthy pregnancy (*χ*^2^_1_=4.9; *P*=.03; question 32); and improve their pregnancy health knowledge (*χ*^2^_1_=16.2; *P*<.001; question 34). For example, frequent Flo app users reported that Flo had helped them to know more about their baby’s development (*χ*^2^_1_=6.6; *P*=.01); their body during pregnancy (*χ*^2^_1_=6.7; *P*=.01); basic do’s and don’ts during pregnancy (*χ*^2^_1_=4.5; *P*=.03; Table S1 in Multimedia Appendix 10, question 36); and how pregnancy affects their physical health (*χ*^2^_1_=4.4; *P*=.04; Table S1 in Multimedia Appendix 10, question 39).

In addition to improvements in pregnancy health and knowledge, study participants who used the Flo app frequently were more likely to report improvements in menstrual health knowledge (Table S2 in Multimedia Appendix 10) such as what symptoms were normal throughout the cycle (*χ*^2^_1_=6.1; *P*=.01; question 47) and how to estimate the most fertile period by using cycle-related knowledge (*χ*^2^_1_=32.7; *P*<.001; question 51). Frequent app users were also more likely to say that Flo had helped them to identify issues related to endometriosis (*χ*^2^_1_=3.9; *P*=.047) and improved communication with their partners about their cycle (*χ*^2^_1_=30.6; *P*<.001; Table S2 in Multimedia Appendix 10, question 47). Finally, frequent Flo app users were more likely to report improvements in communication with a HCPs (*χ*^2^_1_=13.1; *P*<.001; Table S3 in Multimedia Appendix 10, question 53), that is, they became more confident in asking questions about their health and body (*χ*^2^_1_=6.4; *P*=.01) and they became more confident in understanding their reproductive health (*χ*^2^_1_=9.1; *P*=.003; Table S3 in Multimedia Appendix 10, question 64).

Complete responses regarding knowledge and health improvements in pregnancy, cycle, and general health in relation to the frequency of the app use are shown in Tables S1, S2, and S3, respectively, in Multimedia Appendix 10.

### Knowledge and Health Improvements in Relation to Long Versus Short App Use

As shown in Table 4, short-term users reported using the app mainly for help with becoming pregnant (*χ*^2^_1_=26.1; *P*<.001) as opposed to long-term users who reported using the app to help avoid pregnancy (*χ*^2^_1_=30.6; *P*<.001); track menstrual cycle and symptoms (*χ*^2^_1_=46.5; *P*<.001); track irregular cycles and related conditions (*χ*^2^_1_=6.0; *P*=.01); learn more about their body (*χ*^2^_1_=10.3; *P*=.001); learn more about sexual health (*χ*^2^_1_=18.0; *P*<.001); and obtain tailored health information relevant to them (*χ*^2^_1_=8.1; *P*=.004).

We found that short-term Flo users were more likely to report that Flo had helped them to know how to use their cycle to know when they were most fertile (*χ*^2^_1_=8.1; *P*=.008; Table S1 in Multimedia Appendix 11, question 51). At the same time, long-term users were more likely to report improvements across multiple areas of cycle, pregnancy, and general health. Long-term Flo app users were more likely to report that Flo had improved their menstrual health knowledge such as knowing whether their cycle was regular (*χ*^2^_1_=10.0; *P*=.002); cycle length was normal (*χ*^2^_1_=5.4; *P*=.02); and period flow was normal (*χ*^2^_1_=10.3; *P*=.001). In addition, they were more likely to say that Flo had helped them to identify issues related to PCOS (*χ*^2^_1_=6.9; *P*=.009) and helped them to reduce premenstrual syndrome symptoms (*χ*^2^_1_=14.4; *P*<.001; Table S1 in Multimedia Appendix 11, question 47). In addition, long-term users were more likely to say that Flo had helped them to better understand how their cycle affected their mental (*χ*^2^_1_=12.3; *P*<.001) and physical health (*χ*^2^_1_=14.7; *P*<.001) and reactions to situations (lack of patience, sadness, etc; *χ*^2^_1_=9.3; *P*=.002), as well as how to optimize their life around their cycle (*χ*^2^_1_=4.8; *P*=.03; Table S1 in Multimedia Appendix 11, question 51).

In addition, long-term users of Flo reported that had Flo helped them to improve their knowledge of fetal development (*χ*^2^_1_=5.0; *P*=.03) and the management of pregnancy symptoms (*χ*^2^_1_=10.0; *P*=.002) and postpartum symptoms (*χ*^2^_1_=26.0; *P*<.001) as well as the reduction of risk of preterm labor (*χ*^2^_1_=6.1; *P*=.01; Table S2 in Multimedia Appendix 11, question 36 and 39). Finally, long-term users were more likely to report improvements in sexual (*χ*^2^_1_=11.7; *P*<.001); mental (*χ*^2^_1_=12.2; *P*<.001); and physical health (*χ*^2^_1_=13.2; *P*<.001) and health behaviors (*χ*^2^_1_=10.9; *P*<.001; Table S3 in Multimedia Appendix 11, question 53). Most improvements were reported in sexual health knowledge such as STIs and how to avoid them (*χ*^2^_1_=20.9; *P*<.001); the signs and symptoms of STIs (*χ*^2^_1_=16.2; *P*<.001); safe sex (*χ*^2^_1_=14.0; *P*<.001); contraception options (*χ*^2^_1_=12.3; *P*<.001); and the signs and symptoms during sex that were indicative of a health issue (*χ*^2^_1_=8.7; *P*<.003; Table S3 in Multimedia Appendix 11, question 61).

Complete responses regarding knowledge and health improvements in cycle, pregnancy, and general health in relation to short-term versus long-term app use are shown in Tables S1, S2, and S3, respectively, in Multimedia Appendix 11.

## Discussion

### Summary

The aim of this study was to investigate the effects of the Flo app on health and knowledge improvements, with a specific focus on menstrual cycle and pregnancy health. In addition, we aimed to explore whether such effects differed based on education level, country of residence, and app use characteristics (ie, free or paid subscription, frequent or infrequent app use, and short- or long-term app use). We found that most participants reported menstrual cycle and pregnancy knowledge and health improvements upon using the Flo app. These improvements were mostly associated with the use of period and fertile day predictions, symptom-tracking features, and the consumption of relevant in-app health content (articles and videos). Users with different levels of education, countries of residence, and app use patterns differed in their selected health goals. The strongest improvements in knowledge and health were observed in premium, frequent, and long-term users.

### Benefits of Using Menstrual Cycle– and Pregnancy-Tracking Apps

Our study is the first to date examining the effect of the mobile app Flo in improving menstrual and pregnancy knowledge and health as self-reported by its users. Most users who tracked their menstrual cycle with Flo reported that the app had helped them understand whether their menstrual cycle was normal or not; specifically, whether their cycle was regular, whether their cycle length was within the norms, and whether it was typical to have certain symptoms during their cycle. Menstrual cycle is a vital indicator of women’s health, and cycle abnormalities might be a sign of an underlying health issue [52]. For example, prolonged cycle length and cycle irregularity, in combination with other symptoms such as excessive body hair and acne, might be signs of PCOS [38], while painful and heavy periods or bleeding between periods might be indicative of endometriosis [53]. By improving the understanding of what a normal menstrual cycle is and what body symptoms and signs are associated with it, individuals are equipped with tools to make informed decisions as to whether symptoms they experience may require medical attention. Studies show that increased self-awareness promotes successful health behavior changes and makes individuals more proactive about seeking appropriate care when needed [31,54]. This is of vital importance given that the diagnosis of certain conditions such as endometriosis can be delayed for 8 to 12 years [53]. Prevention or early detection of reproductive conditions can improve women’s quality of life as well as decrease the time to diagnosis and medical costs associated with treatment, thereby reducing the economic burden on health care systems [55,56].

One in 3 participants in our sample reported that the Flo app had helped them to improve communication with an HCP. This is not surprising considering that better self-awareness and understanding of one’s own menstrual cycle and body were shown to be important for improving provider-patient communication [31]. According to the UK Women’s Health Strategy Survey, 1 in 4 women do not feel comfortable talking to health care professionals about their menstrual cycle, while most women do not feel heard by their physician [57]. Our results suggest that women’s health apps could improve the education and self-awareness of one’s menstrual health and facilitate patient-physician conversations.

Our survey respondents also reported that the use of the Flo app had helped them manage their menstrual symptoms such as bad mood or cramps. Symptoms that women experience during their menstrual cycle can negatively impact their quality of life, work productivity, and increase health care costs [58]. Dysmenorrhea (a commonly reported cramp-like pain occurring before or during periods) can affect approximately 45% to 95% of women and is associated with productivity loss and absenteeism (ie, absence from work) [59-62]. Furthermore, symptoms of endometriosis such as painful periods or chronic pelvic pain can also negatively impact work performance and can lead to 10.8 hours of lost work per week with an annual total productivity loss of €6298 (US $6838) per woman [55,63], in addition to the profound effects on psychological and social well-being [64,65]. In addition, 65.1% (309/475) of the Flo users reported that the app had helped them manage their irregular cycles and related conditions, with 1 in 3 participants reporting improvements in their mental health. Perceived improvements in cycle symptom management were largely associated with period predictions (935/1276, 73.3%) and reading and watching articles and video sources in the app (787/1276, 61.7%). Given that menstrual cycle symptoms have an impact on several aspects of women’s lives, cycle-tracking apps can help with being mentally prepared for upcoming periods (eg, via period predictions) and can provide insights into how to manage cycle symptoms (eg, via educational content).

Most participants who had used Flo to track their pregnancy reported improvements in pregnancy and postnatal health knowledge (eg, knowing how to manage pregnancy symptoms and which symptoms require medical attention) and well-being (eg, body image issues associated with pregnancy). Women’s health during pregnancy has profound effects on subsequent generations [66]. Poor maternal health can lead to low neonatal weight and reduced chances of survival, congenital abnormalities, problems with child behavior, poor school performance, and adult health and productivity [67-69]. Furthermore, maternal behaviors such as smoking or alcohol consumption during pregnancy may be significant drivers of infant health issues [70,71]. Therefore, health apps that allow pregnancy tracking are promising tools for improving knowledge about maternal and newborn health, thus decreasing the chances of pregnancy-related complications and child health issues.

### Benefits of Using Menstrual Cycle–Tracking Apps for LMICs and HICs

Although participants from LMICs (699/2212, 32.6% of the sample) intended to use the Flo app to improve their sexual health (eg, gaining knowledge of STIs and how to avoid them, safe sex, etc), they also reported significantly higher improvements in multiple areas of their health (menstrual cycle, mental and physical health, and improved healthy behaviors). Compared with HICs, where reproductive and sexual education is mandatory in many schools and menstrual health has started to gain increasing attention from governmental and health care authorities (eg, Women’s Health Strategy in England in 2021) [72], LMICs often lack formal menstrual health education, resulting in high levels of stigma and shame surrounding menstruation and sex [73]. Poor period education results in a lack of knowledge about menstrual cycle norms; menstrual hygiene; gynecological conditions and their symptoms; and sexual health (eg, contraception and STIs). Specifically, according to the World Bank classification, LMICs have the highest prevalence of STIs (eg, gonorrhea, trichomoniasis, and syphilis), and the awareness of STIs other than HIV in those countries is very low [74-76]. Therefore, menstrual and sexual health apps, together with systemic governmental and societal education efforts, can mitigate long-term repercussions of poor health literacy, thereby decreasing unfavorable health outcomes such as infertility, abortions, preterm delivery, and perinatal and neonatal morbidities and helping with cutting health care costs associated with STI treatment [77-80].

Flo app users from HICs reported using the app to help them get pregnant and for pregnancy and menstrual cycle tracking. We found that users from HICs were statistically more likely to report that they became pregnant while using the Flo app, while 97.6% (451/462) acknowledged that fertile days and ovulation predictions made by the Flo app helped them become pregnant. Despite an increase in social and governmental momentum to improve menstrual and sexual health literacy in HICs, topics such as fertility and healthy pregnancy have not received the attention they deserve. A survey conducted on a sample of 1000 American participants showed that younger women (aged 18-24 years) demonstrated a low level of knowledge about conception, ovulation, and the effect of age on the length of time to conception and miscarriage risks. Women aged 25 to 40 years were more likely to believe in common myths around fertility and conception. Finally, more than one-quarter of all survey participants were unaware of factors impacting fertility such as STIs or obesity [18]. Therefore, health apps might help to save costs associated with infertility treatments by educating users on factors impacting fertility and providing fertile window– and ovulation-tracking functionalities to optimize conception strategies.

Menstrual cycle– and pregnancy-tracking apps might represent promising tools for improving reproductive health knowledge and health among women globally. This is largely owing to the anonymity, scalability, and accessibility of such digital solutions. According to a 2019 survey by the Kaiser Family Foundation, 1 in 3 women of reproductive age in the United States used a menstrual cycle–tracking app [81]. At the same time, an increasing adoption of mobile health apps is also observed in LMICs [82]. Thus, app developers and governmental and health authorities should collaborate to promote HCPs encouraging the adoption of menstrual cycle–tracking apps by the target group and facilitate the distribution of health apps in LMICs.

### Modes (Free vs Paid), Frequency, and Length of App Use

We found that participants who used a paid version of the app, as well as those who used the app more frequently and for a longer period, were more likely to report improvements in knowledge and health compared with free app users and infrequent or short-term users. These results are not surprising, as studies show that health interventions that are used consistently for prolonged periods are more likely to positively impact behavior, thus leading to better health outcomes [83]. For example, a study conducted by Huberty et al [84] found that more frequent use of the meditation app Calm was associated with an increase in the likelihood of noticing changes in mental health, sleep, and stress levels. Another study by Han and Rhee [85] showed that users who frequently monitored their weight, food consumption, and exercise via a weight loss app, Noom, had more efficient weight loss over time. Therefore, health app developers should consider designing user-friendly, easy-to-navigate, and evidence-based technologies to encourage higher and consistent engagement with the app to achieve better health outcomes for the users.

### Limitations

Although this study is the first to provide insights into the benefits of using Flo app for women’s health and education, it has several limitations. First, most of the study cohort consisted of highly engaged (1650/2212, 74.6% use the Flo app several times a day to several times a week) and loyal (1305/2212, 59% use the Flo app for >1 year) users of the Flo app who may have more favorable opinions about the app and have benefited more from using it. Those who did not find Flo helpful may have elected not to participate.

Another limitation is that this was a cross-sectional, nonexperimental study that does not allow us to infer causality with regard to the impact of Flo app on user knowledge, health, and well-being. Future randomized controlled trials assessing the efficacy of the Flo app with baseline and follow-up measures should explore these relationships prospectively to determine the extent to which changes can be attributed to Flo app use. Furthermore, systematic exploration of the health benefits of available women’s health apps is needed to allow clinicians and end users to make informed decisions about effective, safe, and trustworthy solutions. To this end, app developers should conduct and make publicly available pre- and postmarket research assessing the effectiveness and efficacy of their health products. Simultaneously, external organizations should conduct rigorous comparisons of such products to facilitate end users’ understanding of benefits and risks. As mentioned in the Introduction section, despite the call for more rigorous evidence generation, research on this topic is scarce, and a more systematic effort is needed across the board.

Finally, the survey questions were specifically designed for this study; thus, they had not been previously validated in scientific research. This study used self-reported statements about perceived health knowledge and health behaviors. Although perceived health knowledge and behaviors do not equate to actual improvements in health literacy and health outcomes, they provide useful preliminary insights into the awareness of one’s own health. Future studies should extend the findings of this report by using validated questionnaires to assess changes in menstrual and pregnancy health literacy, as well as knowledge assessment quizzes over time.

### Conclusions

This study provides the first insights into the effectiveness of the menstrual cycle and women’s health app, Flo, in improving user knowledge and health, and it builds toward a much-needed body of evidence in digital health. Our results highlight the opportunity for menstrual health apps to become useful tools for promoting reproductive health education and empowerment among users globally.

## Appendix

Multimedia Appendix 1 Survey questions.

Multimedia Appendix 2 Flo users’ app use characteristics.

Multimedia Appendix 3 List of high-income countries and total number of users.

Multimedia Appendix 4 List of low- and middle-income countries and total number of users.

Multimedia Appendix 5 Cycle, pregnancy, and general health responses.

Multimedia Appendix 6 Reasons for using the app versus age and education.

Multimedia Appendix 7 Education level versus cycle, pregnancy, and general health questions.

Multimedia Appendix 8 High-income and low- and middle-income countries versus cycle, pregnancy, and general health questions.

Multimedia Appendix 9 Premium and free users versus cycle, pregnancy, and general health questions.

Multimedia Appendix 10 Frequent and infrequent app use versus cycle, pregnancy, and general health questions.

Multimedia Appendix 11 Long- and short-term app use versus cycle, pregnancy, and general health questions.

## Abbreviations

HCP: health care provider
HIC: high-income country
LMIC: low- and middle-income country
PCOS: polycystic ovary syndrome
STI: sexually transmitted infection
