# Supplementary material for: Characterization of Self-reported Improvements in Knowledge and Health Among Users of Flo Period Tracking App: Cross-sectional Survey
Source: JMIR Mhealth Uhealth. 2023 Apr 26;11:e40427. doi: 10.2196/40427 (PMC10173043; doi:10.2196/40427)
Supplement: Multimedia Appendix 1 [file mhealth_v11i1e40427_app1.pdf]

Multimedia Appendix 1. Survey questions.

App Usage Characteristics

* 2. How long have you been using the Flo app?

- Up to one month
- 1–3 months
- 4–6 months
- 7–12 months
- More than a year
- I don’t remember

* 3. How often, on average, do you use the Flo app?

- Several times a day
- Several times a week
- Once a week
- 1–2 times per month
- 1–2 times every three months
- 1–2 times every six months
- 1–2 times per year
- Other (please specify)

* 4. Do you use the free or Premium version of the Flo app?

- Free
- Premium
- I used to use Flo Premium, but not anymore
- I don't know

* 5. How long have you been paying for a Flo subscription?

- Up to one month
- 1–3 months
- 4–6 months
- 7–12 months
- More than a year
- I don’t remember

* 6. How did you sign up for Flo Premium?

- ◯ I signed up after a free trial
- ◯ I signed up for Flo Premium without a trial period
- ◯ I don’t remember

Demographics

* 7.What country do you live in?

* 8.What is your age?

- Under 18
- 18–24
- 25–34
- 35–44
- 45–54
- 55+

* 9.How would you describe yourself?

- American Indian or Alaskan Native
- Asian or Asian-American
- Native Hawaiian or Other Pacific Islander
- White, European-American, or Caucasian
- Other (please specify)
- Black or African American
- Hispanic, Latino, or Spanish origin
- Biracial or Multiracial
- I prefer not to say

* 10. What is your gender identity? (Choose all that apply)

- Woman
- Non-binary
- Genderqueer or gender fluid
- Man
- Questioning or unsure
- Additional gender category/identity not listed (Please specify)
- Trans man
- Trans woman
- Agender
- I prefer not to say

* 11. What is your household income?

- Less than $20,000 per year
- $21,000–40,000
- $41,000–60,000
- $61,000–80,000
- $81,000–100,000
- $101,000–150,000
- $151,000–200,000
- $201,000–250,000
- $250,000 or more per year
- I prefer not to say

* 12. What is the highest level of education you've completed?

- ◯ Some high school, no diploma
- ◯ High school graduate, diploma, or the equivalent (e.g., GED)
- ◯ Associate's degree
- ◯ Bachelor's degree
- ◯ Master's degree
- ◯ Doctorate degree
- ◯ I prefer not to say
- ◯ Other (please specify)

* 13. How important is the following for you?

	Not very important	Somewhat important		Very important	Extremely important
Reproductive health		◯	◯		◯
Mental well-being	◯	◯	◯	◯	◯
Physical well-being	◯	◯	◯	◯	◯
Sexual well-being	◯	◯	◯	◯	◯

Aim of the Flo App Use

* 14. Why do you use Flo?

- [ ] Menstrual cycle and symptom tracking
- [ ] Irregular cycle/ related conditions (e.g., PCOS, endometriosis)
- [ ] Pregnancy tracking
- [ ] To help me to get pregnant
- [ ] To help me not get pregnant
- [ ] Pregnancy loss (e.g., stillbirth, miscarriage, abortion)
- [ ] Sexual health
- [ ] Get tailored female health information relevant to me
- [ ] To learn more about my body
- [ ] Other (please specify)

Cycle Health

* 15. What exactly do you track during your cycle? (Choose all that apply)

- [ ] Menstrual cycle dates and regularity
- [ ] Fertile window and ovulation
- [ ] Cycle signs and symptoms
- [ ] Overall health
- [ ] Contraception
- [ ] Best time for conception
- [ ] Other (please specify)

* 16. Do you believe Flo has helped you manage your menstrual symptoms?

- ( ) Yes
- ( ) No
- ( ) I don’t have any menstrual symptoms

* 17. Which menstrual symptoms has Flo helped you manage? (Choose all that apply)

- [ ] Cramps
- [ ] Tender breasts
- [ ] Acne
- [ ] Backache
- [ ] Nausea
- [ ] Fatigue
- [ ] Bloating
- [ ] Cravings
- [ ] Insomnia
- [ ] Headache
- [ ] Constipation
- [ ] Diarrhea
- [ ] Bad mood (e.g., sad, irritated, feeling guilty, obsessive thoughts, apathetic, confused, or very self-critical)
- [ ] Mood swings
- [ ] Depression
- [ ] Anxiety
- [ ] Discharge
- [ ] Ovulation
- [ ] Other (please specify)

* 18. How much has Flo helped you manage each of these symptoms?

	Not at all	A little	A moderate amount	A lot	A great deal
Cramps	○	○	○	○	○
Tender breasts	○	○	○	○	○
Acne	○	○	○	○	○
Backache	○	○	○	○	○
Nausea	○	○	○	○	○
Fatigue	○	○	○	○	○
Bloating	○	○	○	○	○
Cravings	○	○	○	○	○
Insomnia	○	○	○	○	○
Headache	○	○	○	○	○
Constipation	○	○	○	○	○
Diarrhea	○	○	○	○	○
Bad mood (e.g., sad, irritated, feeling guilty, obsessive thoughts, apathetic, confused, or very self-critical)	○	○	○	○	○
Mood swings	○	○	○	○	○
Depression	○	○	○	○	○
Anxiety	○	○	○	○	○
Discharge	○	○	○	○	○
Ovulation	○	○	○	○	○
[Insert text from Other]	○	○	○	○	○

* 19. What part of using Flo has helped you manage your menstrual symptoms? (Choose all that apply)

- ☐ Reading/watching articles and video sources in the app
- ☐ Symptom- and cycle-related chats with Flo’s chatbot
- ☐ Discussion in Secret Chats
- ☐ Courses with experts
- ☐ Period predictions
- ☐ Fertile days and ovulation predictions
- ☐ Symptom tracking
- ☐ Other (please specify)

* 20. How much these parts on the app have helped you to manage your symptoms?

	Not at all	A little	A moderate amount	A lot	A great deal
Reading/watching articles and video sources in the app	◯	◯	◯	◯	◯
Symptom- and cycle-related chats with Flo's chatbot	◯	◯	◯	◯	◯
Discussion in Secret Chats	◯	◯	◯	◯	◯
Courses with experts	◯	◯	◯	◯	◯
Period predictions	◯	◯	◯	◯	◯
Fertile days and ovulation predictions	◯	◯	◯	◯	◯
Symptom tracking	◯	◯	◯	◯	◯
[Insert text from Other]	◯	◯	◯	◯	◯

Irregular cycle/ related conditions — (e.g., PCOS, or endometriosis)

* 21. Do you believe Flo has helped you manage your condition and symptoms?

- ◯ Yes
- ◯ No

* 22. How helpful has Flo been in helping your condition improve?

- ◯ Not at all helpful
- ◯ Not so helpful
- ◯ Somewhat helpful
- ◯ Very helpful
- ◯ Extremely helpful

* 23. What in the app has helped your condition improve? (Choose all that apply)

- ☐ Reading/watching articles and video sources in the app
- ☐ Symptom- and cycle-related chats with Flo's chatbot
- ☐ Discussion in Secret Chats
- ☐ Courses with experts
- ☐ Period predictions
- ☐ Fertile days and ovulation predictions
- ☐ Symptom tracking
- ☐ Other (please specify)

Irregular cycle/ related conditions — (e.g., PCOS, or endometriosis)

* 24. Do you believe Flo helped you see your health care provider for your condition?

- ○ Yes
- ○ No

* 25. How much has Flo helped your decision to see a health care provider?

- ○ Not at all
- ○ A little
- ○ A moderate amount
- ○ A lot
- ○ A great deal

* 26. What in the app helped your decision to visit a healthcare provider? (Choose all that apply)

- ☐ Reading/watching articles and video sources in the app
- ☐ Symptom- and cycle-related chats with Flo's chatbot
- ☐ Discussion in Secret Chats
- ☐ Courses with experts
- ☐ Period predictions
- ☐ Fertile days and ovulation predictions
- ☐ Symptom tracking
- ☐ Other (please specify)

* 27. How much have these parts of the app helped your condition improve?

	Not at all	A little	A moderate amount	A lot	A great deal
Reading/watching articles and video sources in the app	◯	◯	◯	◯	◯
Symptom- and cycle-related chats with Flo’s chatbot	◯	◯	◯	◯	◯
Discussion in Secret Chats	◯	◯	◯	◯	◯
Courses with experts	◯	◯	◯	◯	◯
Period predictions	◯	◯	◯	◯	◯
Fertile days and ovulation predictions	◯	◯	◯	◯	◯
Symptom tracking	◯	◯	◯	◯	◯
[Insert text from Other]	◯	◯	◯	◯	◯

* 28. How much have these parts of the app helped your decision to visit a health care provider?

	Not at all	A little	A moderate amount	A lot	A great deal
Reading/watching articles and video sources in the app	◯	◯	◯	◯	◯
Symptom- and cycle-related chats with Flo's chatbot	◯	◯	◯	◯	◯
Discussion in Secret Chats	◯	◯	◯	◯	◯
Courses with experts	◯	◯	◯	◯	◯
Period predictions	◯	◯	◯	◯	◯
Fertile days and ovulation predictions	◯	◯	◯	◯	◯
Symptom tracking	◯	◯	◯	◯	◯
[Insert text from Other]	◯	◯	◯	◯	◯

Falling Pregnant and Pregnancy Health

* 29. Did you get pregnant while using Flo?

- ◯ Yes
- ◯ No

* 30. Do you believe that using Flo helped you get pregnant?

- ◯ Yes
- ◯ No

* 31. What in the app helped you get pregnant? (Choose all that apply)

- ☐ Reading/watching articles and video sources in the app
- ☐ Symptom- and cycle-related chats with Flo's chatbot
- ☐ Discussion in Secret Chats
- ☐ Courses with experts
- ☐ Period predictions
- ☐ Fertile days and ovulation predictions
- ☐ Symptom tracking
- ☐ Other (please specify)

* 32. Do you believe Flo helped you prepare for a healthy pregnancy?

- ◯ Yes
- ◯ No

* 33. Do you believe Flo has helped you to be better prepared for natural labor?

- ◯ Yes
- ◯ No

* 34. Do you believe Flo has improved how informed/educated you feel about your pregnancy?

- ◯ Yes
- ◯ No

* 35. How much did these parts of the app help you get pregnant?

	Not at all	A little	A moderate amount	A lot	A great deal
Reading/watching articles and video sources in the app	○	○	○	○	○
Symptom- and cycle-related chats with Flo's chatbot	○	○	○	○	○
Discussion in Secret Chats	○	○	○	○	○
Courses with experts	○	○	○	○	○
Period predictions	○	○	○	○	○
Fertile days and ovulation predictions	○	○	○	○	○
Symptom tracking	○	○	○	○	○
[Insert text from Other]	○	○	○	○	○

* 36. What knowledge about your pregnancy has Flo improved? (Choose all that apply)

- [ ] I know more about baby development
- [ ] I know more about my body during pregnancy
- [ ] I know more of the basic do's and don'ts
- [ ] I know more about what symptoms are OK or not during pregnancy
- [ ] I know more about diet and nutrition during pregnancy
- [ ] I know more about prenatal care
- [ ] Other (please specify)

____

- [ ] None of the above

* 37. How much has Flo improved how informed you feel?

	Not at all	A little	A moderate amount	A lot	A great deal
I know more about baby development	○	○	○	○	○
I know more about my body during pregnancy	○	○	○	○	○
I know more of the basic do's and don'ts	○	○	○	○	○
I know more about what symptoms are OK or not during pregnancy	○	○	○	○	○
I know more about diet and nutrition during pregnancy	○	○	○	○	○
I know more about prenatal care	○	○	○	○	○
[Insert text from Other]	○	○	○	○	○

* 38. What part of using Flo has helped you feel most informed? (Choose all that apply)

- ☐ Reading/watching articles and video sources in the app
- ☐ Symptom- and cycle-related chats with Flo's chatbot
- ☐ Discussion in Secret Chats
- ☐ Courses with experts
- ☐ Period predictions
- ☐ Fertile days and ovulation predictions
- ☐ Symptom tracking
- ☐ Other (please specify)

____

* 39. Which do you believe Flo has helped you better understand? (Choose all that apply)

- [ ] How to manage your pregnancy symptoms
- [ ] How your pregnancy affects your mental health (feeling sad, feeling anxious, mood swings, etc.)
- [ ] How your pregnancy affects your physical health (fatigue, constipation, bloating, etc.)
- [ ] How your pregnancy affects your reaction to situations (impatience, etc.)
- [ ] How to optimize your life around your pregnancy (exercise/physical activity, sleep, sex life, etc.)
- [ ] How to manage your postpartum symptoms
- [ ] How to reduce your risk for preterm labor
- [ ] Other (please specify)
- [ ] None of the above

* 40. How much has your understanding improved?

	Not at all	A little	A moderate amount	A lot	A great deal
How to manage your pregnancy symptoms	○	○	○	○	○
How your pregnancy affects your mental health (feeling sad, feeling anxious, mood swings, etc.)	○	○	○	○	○
How your pregnancy affects your physical health (fatigue, constipation, bloating, etc.)	○	○	○	○	○
How your pregnancy affects your reaction to situations (impatience, etc.)	○	○	○	○	○
How to optimize your life around your pregnancy (exercise/physical activity, sleep, sex life, etc.)	○	○	○	○	○
How to manage your postpartum symptoms	○	○	○	○	○
How to reduce your risk for preterm labor	○	○	○	○	○
[Insert text from Other]	○	○	○	○	○

* 41. How long have you been trying to conceive?

- ○ 0–6 months
- ○ 6–12 months
- ○ 1–2 years
- ○ Longer than 2 years

* 42. Do you believe Flo has helped you to conceive faster?

- ○ Yes
- ○ No
- ○ I am still trying

* 43. What in the app has helped you to conceive faster? (Choose all that apply)

- ☐ Reading/watching articles and video sources in the app
- ☐ Symptom- and cycle-related chats with Flo's chatbot
- ☐ Discussion in Secret Chats
- ☐ Courses with experts
- ☐ Period predictions
- ☐ Fertile days and ovulation predictions
- ☐ Symptom tracking
- ☐ Other (please specify)

____

* 44. How much did these parts of the app help you to conceive faster?

	Not at all	A little	A moderate amount	A lot	A great deal
Reading/watching articles and video sources in the app	○	○	○	○	○
Symptom- and cycle-related chats with Flo's chatbot	○	○	○	○	○
Discussion in Secret Chats	○	○	○	○	○
Courses with experts	○	○	○	○	○
Period predictions	○	○	○	○	○
Fertile days and ovulation predictions	○	○	○	○	○
Symptom tracking	○	○	○	○	○
[Insert text from Other]	○	○	○	○	○

* 45. Do you believe Flo has helped you to avoid fertility treatments (IVF, ICSI, etc.)

- ◯ Yes
- ◯ No

Cycle Education

* 46. Do you believe Flo has improved how informed/educated you feel about your cycle health?

- ◯ Yes
- ◯ No

* 47. What knowledge about your cycle health has Flo improved? (Choose all that apply)

- ☐ I know my cycle is regular/irregular
- ☐ I know my cycle length is normal/abnormal
- ☐ I know my period flow is normal/abnormal
- ☐ I know I am/am not ovulating
- ☐ I know it’s normal/not normal to have certain symptoms throughout my cycle (period cramps, spotting, headaches, etc.)
- ☐ Fo helped me identify whether my vaginal discharge is healthy throughout my cycle Flo
- ☐ encouraged me to see a doctor, and I’ve been diagnosed with a condition Flo helped me identify
- ☐ issues related to PCOS
- ☐ Flo helped me identify issues related to endometriosis
- ☐ Flo helped me identify issues related to perimenopause
- ☐ Flo helped me decrease the negative effects of PMS
- ☐ Flo helped me communicate more with my partner about my cycle Other
- ☐ (please specify)

* 48. How much has Flo improved how informed you feel?

- ◯ Not at all
- ◯ A little
- ◯ A moderate amount
- ◯ A lot
- ◯ A great deal

* 49. What part of using Flo has helped you feel most informed? (Choose all that apply)

- [ ] Reading/watching articles and video sources in the app
- [ ] Symptom- and cycle-related chats with Flo's chatbot
- [ ] Discussion in Secret Chats
- [ ] Courses with experts
- [ ] Period predictions
- [ ] Fertile days and ovulation predictions
- [ ] Symptom tracking
- [ ] Other (please specify)

* 50. How much did using these parts of the app improve how informed you feel about your cycle health?

	Not at all	A little	A moderate amount	A lot	A great deal
Reading/watching articles and video sources in the app	○	○	○	○	○
Symptom- and cycle-related chats with Flo's chatbot	○	○	○	○	○
Discussion in Secret Chats	○	○	○	○	○
Courses with experts	○	○	○	○	○
Period predictions	○	○	○	○	○
Fertile days and ovulation predictions	○	○	○	○	○
Symptom tracking	○	○	○	○	○
[Insert text from Other]	○	○	○	○	○

* 51. Which do you believe Flo has helped you better understand? (Choose all that apply)

- [ ] How to manage your menstrual cycle symptoms
- [ ] How your cycle affects your mental health (feeling sad, mood swings, etc.)
- [ ] How your cycle affects your physical health (tired, bloated, etc.)
- [ ] How your cycle affects your reaction to situations (lack of patience, sadness, etc.)
- [ ] How to use your cycle to know when you are most fertile
- [ ] How to optimize your life around your cycle (exercise/physical activity, sleep, sex life, etc.)
- [ ] Other (please specify)
- [ ] None of the above

* 52. How much has your understanding improved?

	Not at all	A little	A moderate amount	A lot	A great deal
How to manage your menstrual cycle symptoms	○	○	○	○	○
How your cycle affects your mental health (feeling sad, mood swings, etc.)	○	○	○	○	○
How your cycle affects your physical health (tired, bloated, etc.)	○	○	○	○	○
How your cycle affects your reaction to situations (lack of patience, sadness, etc.)	○	○	○	○	○
How to use your cycle to know when you are most fertile	○	○	○	○	○
How to optimize your life around your cycle (exercise/physical activity, sleep, sex life, etc.)	○	○	○	○	○
[Insert text from Other]	○	○	○	○	○

General Health

* 53. Do you believe that using Flo has helped you improve any of the following?

- [ ] Mental health and emotional well-being (reducing stress, being happier, etc.)
- [ ] Physical health (feeling less tired, improving skin, etc.)
- [ ] Health behaviors (improved diet, increased activity, etc.)
- [ ] Sexual health (improved sex life, safe sex, etc.)
- [ ] Communication with your health care provider
- [ ] None of the above

* 54. How much has Flo helped you to improve the following?

	Not at all	A little	A moderate amount	A lot	A great deal
Mental health and emotional well-being (reducing stress, being happier, etc.)	○	○	○	○	○
Physical health (feeling less tired, improving skin, etc.)	○	○	○	○	○
Health behaviors (improved diet, increased activity, etc.)	○	○	○	○	○
Sexual health (improved sex life, safe sex, etc.)	○	○	○	○	○
Communication with your health care provider	○	○	○	○	○

General Health: Mental Health and Emotional Well-being

* 55. What aspects of your mental health and emotional well-being do you think Flo has helped you with and to what extend? (Choose all that apply)

	Not at all	A little	A moderate amount	A lot	A great deal
Reducing stress	○	○	○	○	○
Reducing anxiety and worry	○	○	○	○	○
Managing low mood and depressive symptoms	○	○	○	○	○
Improving your ability to control your emotions	○	○	○	○	○
Being happier	○	○	○	○	○
Having better emotional well-being	○	○	○	○	○
Improving your self-esteem	○	○	○	○	○
Improving your confidence	○	○	○	○	○
Improving relationships with others	○	○	○	○	○

Other (please specify)

* 56. What part of using Flo has helped you improve your mental health and well-being and to what extend? (Choose all that apply)

	Not at all	A little	A moderate amount	A lot	A great deal
Reading/watching articles and video sources in the app	○	○	○	○	○
Symptom- and cycle-related chats with Flo's chatbot	○	○	○	○	○
Discussion in Secret Chats	○	○	○	○	○
Courses with experts	○	○	○	○	○
Period predictions	○	○	○	○	○
Fertile days and ovulation predictions	○	○	○	○	○
Symptom tracking	○	○	○	○	○

General Health: Physical Health

* 57. What aspects of your physical health do you think Flo has helped you with and to what extend? (Choose all that apply)

	Not at all	A little	A moderate amount	A lot	A great deal
Feeling less fatigued and tired	○	○	○	○	○
Having more energy	○	○	○	○	○
Sleeping better	○	○	○	○	○
Managing your weight	○	○	○	○	○
Improving your skin	○	○	○	○	○
Improving your fitness	○	○	○	○	○

Other (please specify)

* 58. What part of using Flo has helped you improve your physical health and to what extend? (Choose all that apply)

	Not at all	A little	A moderate amount	A lot	A great deal
Reading/watching articles and video sources in the app	○	○	○	○	○
Symptom- and cycle-related chats with Flo's chatbot	○	○	○	○	○
Discussion in Secret Chats	○	○	○	○	○
Courses with experts	○	○	○	○	○
Period predictions	○	○	○	○	○
Fertile days and ovulation predictions	○	○	○	○	○
Symptom tracking	○	○	○	○	○

General Health: Health Behaviors

* 59. What aspects of your health behaviors do you think Flo has helped you with and to what extend? (Choose all that apply)

	Not at all	A little	A moderate amount	A lot	A great deal
Increased activity/exercise	◯	◯	◯	◯	◯
Improved diet	◯	◯	◯	◯	◯
Better stress management (e.g., meditation, breathing exercises, and journaling)	◯	◯	◯	◯	◯
Reducing harmful habits	◯	◯	◯	◯	◯
Better management of symptoms (cramps, bloating, etc.)	◯	◯	◯	◯	◯

Other (please specify)

* 60. What part of using Flo has helped you improve your health behaviors and to what extend? (Choose all that apply)

	Not at all	A little	A moderate amount	A lot	A great deal
Reading/watching articles and video sources in the app	◯	◯	◯	◯	◯
Symptom- and cycle-related chats with Flo's chatbot	◯	◯	◯	◯	◯
Discussion in Secret Chats	◯	◯	◯	◯	◯
Courses with experts	◯	◯	◯	◯	◯
Period predictions	◯	◯	◯	◯	◯
Fertile days and ovulation predictions	◯	◯	◯	◯	◯
Symptom tracking	◯	◯	◯	◯	◯

General Health: Sexual Health

* 61. What aspects of your sexual health do you think Flo has made you more informed about and to what extend? (Choose all that apply)

	Not at all	A little	A moderate amount	A lot	A great deal
STIs and how to avoid them	○	○	○	○	○
The signs and symptoms of STIs	○	○	○	○	○
Safe sex	○	○	○	○	○
Contraception options	○	○	○	○	○
The signs and symptoms during sex that are indicative of a health issue	○	○	○	○	○

Other (please specify)

* 62. What aspects of your sexual health do you think Flo has helped you achieve and to what extend? (Choose all that apply)

	Not at all	A little	A moderate amount	A lot	A great deal
Improved sex life	○	○	○	○	○
More pleasure during sex	○	○	○	○	○
Better understanding of your sexuality	○	○	○	○	○
Getting rid of sex-related stigmas	○	○	○	○	○
Improved self-love and self-compassion	○	○	○	○	○

Other (please specify)

General Health: Sexual Health

* 63. What part of using Flo has helped you improve your sexual health and to what extend? (Choose all that apply)

	Not at all	A little	A moderate amount	A lot	A great deal
Reading/watching articles and video sources in the app	○	○	○	○	○
Symptom- and cycle-related chats with Flo's chatbot	○	○	○	○	○
Discussion in Secret Chats	○	○	○	○	○
Courses with experts	○	○	○	○	○
Period predictions	○	○	○	○	○
Fertile days and ovulation predictions	○	○	○	○	○
Symptom tracking	○	○	○	○	○

Communication with a Healthcare Provider

* 64. What do you feel more confident about when communicating with your health care provider?

- [ ] I am more confident about asking questions about my health/body
- [ ] I am more confident about asking for resources that I think I need (e.g., stress relief, birth control, support) I am more
- [ ] confident about sharing what is going on with my body
- [ ] I am more confident about my understanding of my reproductive health Other
- [ ] (please specify)

* 65. How much has Flo helped you be more confident on each statement?

	Not at all	A little	A moderate amount	A lot	A great deal
I am more confident about asking questions about my health/body	○	○	○	○	○
I am more confident about asking for resources that I think I need (e.g., stress relief, birth control, support)	○	○	○	○	○
I am more confident about sharing what is going on with my body	○	○	○	○	○
I am more confident about my understanding of my reproductive health	○	○	○	○	○
[Insert text from Other]	○	○	○	○	○

Workplace Productivity and Healthcare Costs

* 66. How helpful has Flo been? (Choose all that apply)

	Not at all	A little	A moderate amount	A lot	A great deal
Preventing work burnout	◯	◯	◯	◯	◯
Increasing your productivity at work	◯	◯	◯	◯	◯
Helping you cope with work-related stress	◯	◯	◯	◯	◯

* 67. Do you have private health insurance?

◯ Yes

◯ No

◯ I prefer not to answer

* 68. Do you believe Flo has helped you to save money on your yearly health care provider visits?

◯ Yes

◯ No

◯ I don't know

* 69. Do you believe Flo has helped you to save money on your yearly emergency room visits?

◯ Yes

◯ No

◯ I don't know

Flo Recommendation

* 70. How likely are you to recommend Flo to friends?

- ◯ Very unlikely
- ◯ Unlikely
- ◯ Neither likely nor unlikely
- ◯ Likely
- ◯ Very likely
