# Supplementary material for: Characterization of Self-reported Improvements in Knowledge and Health Among Users of Flo Period Tracking App: Cross-sectional Survey
Source: JMIR Mhealth Uhealth. 2023 Apr 26;11:e40427. doi: 10.2196/40427 (PMC10173043; doi:10.2196/40427)
Supplement: Multimedia Appendix 2 [file mhealth_v11i1e40427_app2.pdf]

Multimedia Appendix 2. Flo users' app use characteristics.

Question	Frequency, n (%)
**How long have you been using the Flo App?**	
Up to one month	69 (3.1)
1 - 3 Months	190 (8.6)
4 - 6 Months	221 (10.0)
7 - 12 Months	350 (15.8)
More than a year	1305 (59.0)
I don't remember	77 (3.5)
**How often, on average, do you use the Flo App?**	
Several times a day	666 (30.1)
Several times a week	984 (44.5)
Once a week	200 (9.0)
1 - 2 times per month	244 (11.0)
1 - 2 times every three months	14 (0.6)
1 - 2 times every six months	1 (0.05)
1 - 2 times per year	5 (0.2)
Other	98 (4.4)
**Do you use the Free or Premium version?**	
Premium	1079 (48.8)
Free	915 (41.4)
I used to own Premium	154 (7.0)
I don't know	64 (2.9)
**How long have you been paying for a Flo Subscription?**[a]	
Up to one month	151 (12.2)
1 - 3 Months	195 (15.8)
4 - 6 Months	180 (14.6)
7 - 12 Months	381 (30.9)
More than a year	215 (17.4)
I don't remember	128 (10.4)
**How did you sign up to Flo Premium?**[a]	
After free trial	747 (60.6)
Without free trial	277 (22.5)
I don't remember	209 (17.0)
**What do you use the Flo App for?**	
Menstrual Cycle and Symptom Tracking	1794 (81.1)
Help get Pregnant	1191 (53.8)
Learn more about my body	914 (41.3)
Pregnancy Tracking	824 (37.3)
Tailored Health Information	733 (33.1)
Sexual Health	625 (28.3)
Irregular Cycle/Related Conditions	473 (21.4)
Help not get Pregnant	250 (11.3)
Pregnancy Loss	151 (6.8)
**How likely are you to recommend the Flo App to your friends?**	
Very Likely	966 (43.7)
Likely	906 (41.0)
Neither Likely nor unlikely	272 (12.3)
Unlikely	34 (1.5)
Very Unlikely	34 (1.5)

[a] Number of Respondents for these questions was 1,233. As only Premium and Used to be Premium users were asked.
