# Supplementary material for: Characterization of Self-reported Improvements in Knowledge and Health Among Users of Flo Period Tracking App: Cross-sectional Survey
Source: JMIR Mhealth Uhealth. 2023 Apr 26;11:e40427. doi: 10.2196/40427 (PMC10173043; doi:10.2196/40427)
Supplement: Multimedia Appendix 3 [file mhealth_v11i1e40427_app3.pdf]

**Multimedia appendix 3.** List of high-income countries and total number of users.

| High-income country | Number of users |
|---|---|
| United States of America | 758 |
| United Kingdom | 217 |
| Canada | 121 |
| Australia | 92 |
| Netherlands | 32 |
| Ireland | 31 |
| Germany | 30 |
| Czech Republic | 16 |
| Romania | 17 |
| United Arab Emirates | 16 |
| Belgium | 12 |
| Greece | 12 |
| New Zealand | 11 |
| France | 9 |
| Slovenia | 9 |
| Lithuania | 8 |
| Singapore | 8 |
| Hungary | 7 |
| Italy | 7 |
| Spain | 7 |
| Sweden | 7 |
| Barbados | 6 |
| Croatia | 6 |
| Latvia | 6 |
| Poland | 6 |
| Denmark | 5 |
| Finland | 5 |
| Norway | 5 |
| Saudi Arabia | 5 |
| Switzerland | 5 |
| Israel | 4 |
| Malta | 4 |
| Portugal | 4 |
| Iceland | 3 |
| Japan | 3 |
| Luxembourg | 3 |
| Qatar | 3 |
| Austria | 2 |
| Cyprus | 2 |
| Korea Republic | 2 |
| Antigua and Barbuda | 1 |
| British Virgin Islands | 1 |
| Kuwait | 1 |
| Oman | 1 |
| Saint Kitts and Nevis | 1 |

| | |
|---|---|
| Slovakia | 1 |
| Turks and Caicos | 1 |
