# Supplementary material for: Characterization of Self-reported Improvements in Knowledge and Health Among Users of Flo Period Tracking App: Cross-sectional Survey
Source: JMIR Mhealth Uhealth. 2023 Apr 26;11:e40427. doi: 10.2196/40427 (PMC10173043; doi:10.2196/40427)
Supplement: Multimedia Appendix 4 [file mhealth_v11i1e40427_app4.pdf]

Multimedia appendix 4. List of low- and middle-income countries and total number of users.

Low- and middle-income	Number of users
India	108
South Africa	86
Nigeria	82
Ghana	63
Philippines	43
Kenya	26
Pakistan	24
Jamaica	18
Trinidad and Tobago	15
Indonesia	13
Zambia	13
Uganda	12
Sri Lanka	9
Serbia	8
Malaysia	8
Egypt	8
Belarus	7
Bulgaria	7
Zimbabwe	7
Brazil	6
Nepal	6
United Republic of Tanzania	6
Russian Federation	5
Mauritius	5
Mexico	5
Georgia	5
Bosnia and Herzegovina	4
Saint Vincent and the Grenadines	4
Lebanon	4
Suriname	4
Ukraine	4
Bangladesh	4
Iraq	4
Albania	4
Iran	4
Belize	4
Thailand	3
Bahamas	3
Turkey	3
Algeria	3
Jordan	3
Namibia	3
Maldives	3
Brunei Darussalam	2
Sierra Leone	2

Estonia	2
Guyana	2
Gambia	2
Cameroon	2
China	2
Republic of Moldova	2
Haiti	2
Malawi	2
Lesotho	2
Cuba	1
Grenada	1
Democratic Republic of the Congo	1
Saint Lucia	1
Uzbekistan	1
Armenia	1
Honduras	1
Tunisia	1
Morocco	1
Micronesia (Federated States of)	1
Vietnam	1
Somalia	1
Swaziland	1
Liberia	1
Ethiopia	1
Burundi	1
Macedonia	1
Rwanda	1
Bahrain	1
Papua New Guinea	1
Botswana	1
Sudan	1
Azerbaijan	1
Myanmar	1
