# Supplementary material for: Characterization of Self-reported Improvements in Knowledge and Health Among Users of Flo Period Tracking App: Cross-sectional Survey
Source: JMIR Mhealth Uhealth. 2023 Apr 26;11:e40427. doi: 10.2196/40427 (PMC10173043; doi:10.2196/40427)
Supplement: Multimedia Appendix 5 [file mhealth_v11i1e40427_app5.pdf]

Multimedia appendix 5.

Table S1. Cycle health responses.

Question					Response
16	**Yes, n (%)**				**No, n (%)**
	1270 (77.3)				372 (22.7)
17	**Symptom**				**Frequency, n (%)**
	Cramp				618 (48.7)
	Tender Breasts				393 (30.9)
	Acne				312 (24.6)
	Backache				332 (26.1)
	Fatigue				398 (31.3)
	Nausea				224 (17.6)
	Bloating				425 (33.5)
	Insomnia				194 (15.3)
	Cravings				298 (23.5)
	Headache				432 (34.0)
	Constipation				205 (16.1)
	Diarrhoea				143 (11.3)
	Bad Mood				641 (50.5)
	Mood Swings				541 (42.6)
	Depression				224 (17.6)
	Anxiety				333 (26.2)
	Discharge				462 (36.4)
	Ovulation				668 (52.6)
	Other				48 (3.8)
19	**Part of App**				**Frequency, n (%)**
	Reading/watching articles in the app				787 (62.0)
	Chats with Flo's Chatbot				714 (56.2)
	Discussion in Secret Chats				458 (36.1)
	Courses with Experts				256 (20.2)
	Period Predictions				935 (73.6)
	Fertile Days and Ovulation Predictions				797 (62.8)
	Symptom Tracking				784 (61.7)
	Other				9 (0.7)
21	**Yes, n (%)**				**No, n (%)**
	309 (65.1)				166 (34.9)
23	**Part of App**				**n, (%)**
	Reading/watching articles in the app				141 (45.6)
	Chats with Flo's Chatbot				161 (52.1)
	Discussion in Secret Chats				92 (29.8)
	Courses with Experts				69 (22.3)
	Period Predictions				212 (68.6)
	Fertile Days and Ovulation Predictions				167 (54.0)
	Symptom Tracking				189 (61.1)
	Other				5 (1.6)
46	**Yes, n (%)**				**No, n (%)**
	1292 (89.0)				160 (11.0)
47	**Area of Knowledge**				**Frequency, n (%)**
	I know my cycle is regular/irregular				1085 (84.0)
	I know my cycle length is normal/abnormal				978 (75.7)
	I know my period flow is normal/abnormal				651 (50.4)
	I know I am/am not ovulating				713 (55.2)
	I know its normal/not normal to have certain symptoms throughout my cycle				755 (58.4)
	I know if my vaginal discharge is healthy throughout my cycle				596 (46.1)
	Flo encouraged me to see a doctor, and I've been diagnosed with a condition				94 (7.3)
	Identify issues related to PCOS				111 (8.6)
	Identify issues related to endometriosis				58 (4.5)
	Identify issues related to perimenopause				33 (2.6)
	Decrease negative effects of PMS				256 (19.8)
	Helped me communicate more about my cycle to my partner				402 (31.1)
	Other				14 (1.1)
48	**Not at all, n (%)**	**Little, n (%)**	**Moderate, n (%)**	**A lot, n (%)**	**A great deal, n (%)**
	12 (0.9)	175 (13.5)	495 (30.6)	452 (35.0)	258 (20.0)
49	**Part of App**				**Frequency, n (%))**
	Reading/watching articles in the app				654 (50.6)
	Chats with Flo's Chatbot				629 (48.7)
	Discussion in Secret Chats				407 (31.5)
	Courses with Experts				220 (17.0)
	Period Predictions				805 (62.3)
	Fertile Days and Ovulation Predictions				752 (58.2)
	Symptom Tracking				657 (50.9)
	Other				12 (1.0)
51	**Area Better Understood**				**Frequency, n (%)**
	How to manage your menstrual symptoms				603 (46.7)

How your cycle affects mental health	765 (59.2)
How your cycle affects physical health	776 (60.1)
How your cycle affects your reaction to situations	572 (44.3)
How to use your cycle to know when you are most fertile	780 (60.4)
How to optimize your life around your cycle	525 (40.6)
None of the above	56 (4.3)
Other	8 (0.6)

Table S2. Pregnancy responses.

Question		Response
29	**Yes, n (%)**	**No, n (%)**
	627 (76.1)	197 (23.9)
30	**Yes, n (%)**	**No, n (%)**
	461 (73.5)	166 (26.5)
31	**Part of App**	**Frequency, n (%)**
	Reading/watching articles in the app	108 (23.4)
	Chats with Flo's Chatbot	96 (20.8)
	Discussion in Secret Chats	58 (12.6)
	Courses with Experts	47 (10.2)
	Period Predictions	274 (59.4)
	Fertile Days and Ovulation Predictions	451 (97.6)
	Symptom Tracking	191 (41.3)
	Other	4 (0.8)
32	**Yes, n (%)**	**No, n (%)**
	530 (84.5)	97 (15.5)
33	**Yes, n (%)**	**No, n (%)**
	127 (64.5)	70 (35.5)
34	**Yes, n (%)**	**No, n (%)**
	698 (84.7)	126 (15.3)
36	**Knowledge Area**	**Frequency, n (%)**
	Baby Development	577 (82.7)
	Body During Pregnancy	589 (84.4)
	Basic Do's and Don'ts	492 (70.5)
	What symptoms are ok and not during pregnancy	505 (72.3)
	Diet and Nutrition during Pregnancy	395 (56.6)
	Prenatal Care	379 (54.3)
	None of the Above	15 (2.1)
	Other	7 (1.1)
38	**Part of App**	**Frequency, n (%)**
	Reading/watching articles in the app	503 (72.1)
	Chats with Flo's Chatbot	220 (31.5)
	Discussion in Secret Chats	230 (33.0)
	Courses with Experts	199 (28.5)
	Period Predictions	258 (37.0)
	Fertile Days and Ovulation Predictions	356 (51.0)
	Symptom Tracking	245 (35.1)
	Other	9 (1.2)
39	**Better Understand**	**Frequency, n (%)**
	Manage Pregnancy Symptoms	403 (57.7)
	How Pregnancy affects mental health	384 (55.0)
	How Pregnancy affects physical health	509 (72.9)
	How Pregnancy affects reactions	257 (36.8)
	How to optimize your life around your pregnancy	393 (56.3)
	How to manage postpartum symptoms	149 (21.3)
	Reduce risk for preterm labour	167 (23.9)
	None of the Above	62 (8.9)
	Other	3 (0.4)

Table S3. General health responses.

Question	Response					
53	**Area of Improvement**	**Frequency,n (%)**				
	Mental and emotional well-being	843 (38.1)				
	Physical Health	589 (26.6)				
	Health Behaviours	621 (28.1)				
	Sexual Health	835 (37.7)				
	Communication with HCP	587 (26.5)				
	None of the above	669 (30.2)				
54	**Improved Area**	**Not at all, n (%)**	**Little, n (%)**	**Moderate, n (%)**	**A lot, n (%)**	**A great deal, n (%)**
	Mental and emotional well-being	4 (0.5)	109 (12.9)	298 (35.4)	274 (32.5)	158 (18.7)
	Physical Health	3 (0.5)	72 (12.2)	203 (34.6)	188 (31.9)	123 (20.8)
	Health Behaviours	8 (1.3)	73 (11.7)	222 (35.9)	185 (29.7)	133 (21.4)
	Sexual Health	4 (0.5)	93 (11.1)	252 (30.2)	290 (34.6)	199 (23.7)
55	**Mental Health Improvement**	**Not at all, n (%)**	**Little, n (%)**	**Moderate, n (%)**	**A lot, n (%)**	**A great deal, n (%)**
	Reducing Stress	29 (3.4)	171 (20.3)	327 (38.9)	218 (25.8)	98 (11.6)
	Reducing Anxiety and Worry	31 (3.7)	158 (18.7)	312 (37.1)	241 (28.6)	101 (12.0)
	Managing low mood and depressive symptoms	53 (6.3)	187 (22.2)	289 (34.4)	219 (25.9)	95 (11.3)
	Improve your ability to control emotions	72 (8.5)	192 (22.7)	270 (32.1)	211 (25.0)	98 (11.6)
	Being happier	40 (4.7)	196 (23.3)	288 (34.1)	113 (25.2)	106 (12.6)
	Having better emotional well-being	30 (3.6)	156 (18.5)	316 (37.6)	236 (28.0)	105 (12.4)
	Improving self-esteem	81 (9.6)	166 (12.8)	256 (30.3)	214 (25.4)	126 (14.9)
	Improving Confidence	80 (9.5)	166 (19.7)	260 (30.9)	213 (25.2)	124 (14.7)
	Improving Relationships with others	95 (11.3)	190 (22.5)	242 (28.8)	208 (24.6)	108 (12.8)
56	**Part of App**	**Not at all, n (%)**	**Little, n (%)**	**Moderate, n (%)**	**A lot, n (%)**	**A great deal, n (%)**
	Reading/watching articles in the app	70 (8.3)	157 (18.7)	254 (30.0)	212 (25.1)	150 (17.8)
	Chats with Flo's Chatbot	84 (10.0)	153 (18.2)	245 (29.0)	217 (25.7)	144 (18.2)
	Discussion in Secret Chats	196 (23.2)	160 (19.1)	189 (22.4)	160 (19.0)	138 (16.4)
	Courses with Experts	172 (20.4)	185 (22.3)	226 (26.8)	147 (17.4)	113 (13.4)
	Period Predictions	36 (4.3)	98 (11.7)	183 (21.7)	299 (35.4)	227 (26.9)
	Fertile Days and Ovulation Predictions	55 (6.5)	96 (11.5)	185 (21.9)	283 (33.5)	224 (26.5)
	Symptom Tracking	28 (3.3)	107 (12.8)	192 (22.7)	296 (35.1)	220 (26.1)
57	**Physical Health Improvement**	**Not at all, n (%)**	**Little, n (%)**	**Moderate, n (%)**	**A lot, n (%)**	**A great deal, n (%)**
	Feeling less fatigued and tired	40 (6.8)	134 (22.9)	213 (36.1)	133 (22.5)	69 (11.7)
	Having more energy	48 (8.1)	130 (22.2)	212 (35.9)	127 (21.5)	72 (12.2)
	Sleeping Better	53 (9.2)	130 (22.0)	188 (31.9)	139 (23.6)	79 (13.4)
	Managing your weight	105 (17.8)	149 (25.3)	169 (28.8)	198 (16.6)	68 (11.5)
	Improving your skin	92 (14.6)	151 (25.8)	166 (28.1)	111 (18.8)	69 (11.7)
58	**Part of App**	**Not at all, n (%)**	**Little, n (%)**	**Moderate, n (%)**	**A lot, n (%)**	**A great deal, n (%)**
	Reading/watching articles in the app	49 (8.3)	126 (21.4)	169 (28.8)	139 (23.6)	106 (18.0)
	Chats with Flo's Chatbot	67 (11.4)	134 (22.7)	155 (26.4)	142 (24.1)	91 (15.4)
	Discussion in Secret Chats	133 (22.5)	120 (20.3)	145 (24.4)	107 (18.1)	86 (14.6)
	Courses with Experts	116 (19.7)	128 (21.7)	151 (25.8)	110 (18.6)	84 (14.2)
	Period Predictions	39 (6.6)	78 (13.2)	136 (23.2)	193 (32.7)	143 (23.2)
	Fertile Days and Ovulation Predictions	42 (7.1)	76 (12.9)	135 (23.1)	190 (32.2)	146 (24.7)
	Symptom Tracking	18 (3.1)	76 (12.9)	165 (28.1)	185 (31.4)	145 (24.6)
59	**Health Behaviour Improvement**	**Not at all, n (%)**	**Little, n (%)**	**Moderate, n (%)**	**A lot, n (%)**	**A great deal, n (%)**
	Increased Activity/Exercise	74 (11.9)	173 (28.0)	202 (32.5)	115 (18.5)	57 (9.2)
	Improved Diet	59 (9.5)	150 (24.1)	204 (33.0)	140 (22.5)	68 (10.9)
	Better Stress Management	56 (9.0)	153 (24.8)	197 (31.7)	141 (22.7)	74 (11.9)
	Reducing Harmful Habits	87 (14.0)	155 (25.1)	164 (26.4)	164 (22.0)	87 (12.5)
	Better Management of Symptoms	25 (4.0)	120 (19.5)	195 (31.4)	178 (28.6)	103 (16.6)
60	**Part of App**	**Not at all, n (%)**	**Little, n (%)**	**Moderate, n (%)**	**A lot, n (%)**	**A great deal, n (%)**
	Reading/watching articles in the app	40 (6.4)	121 (19.9)	183 (29.4)	166 (26.7)	111 (17.8)
	Chats with Flo's Chatbot	68 (10.9)	135 (21.7)	185 (29.7)	142 (22.7)	91 (14.7)
	Discussion in Secret Chats	143 (23.0)	146 (23.6)	141 (22.7)	103 (16.6)	88 (14.1)
	Courses with Experts	108 (17.4)	141 (22.8)	161 (25.9)	124 (19.9)	87 (14.0)
	Period Predictions	32 (5.1)	80 (12.7)	149 (24.0)	197 (31.7)	165 (26.5)
	Fertile Days and Ovulation Predictions	46 (7.4)	89 (14.5)	148 (23.8)	175 (28.1)	163 (26.2)
	Symptom Tracking	29 (4.7)	82 (13.3)	169 (27.2)	182 (29.3)	159 (25.6)
61	**Sexual Health Improvement**	**Not at all, n (%)**	**Little, n (%)**	**Moderate, n (%)**	**A lot, n (%)**	**A great deal, n (%)**
	STI's and how to avoid them	208 (24.9)	166 (19.9)	176 (21.0)	161 (19.2)	125 (14.9)
	The signs and symptoms of STI's	225 (27.0)	167 (20.0)	161 (19.2)	161 (19.2)	123 (14.7)

	Safe Sex	139 (16.6)	141 (16.9)	189 (22.3)	205 (24.5)	163 (19.5)
	Contraception Options	160 (19.0)	145 (17.4)	185 (22.1)	183 (21.9)	164 (19.6)
	Signs and Symptoms during sex that are indicative of a health issue	127 (15.2)	151 (18.1)	215 (25.6)	187 (22.3)	157 (18.7)
62	**Sexual Health Achieved**	**Not at all, n (%)**	**Little, n (%)**	**Moderate, n (%)**	**A lot, n (%)**	**A great deal, n (%)**
	Improved Sex Life	66 (7.9)	162 (19.5)	160 (31.1)	207 (24.7)	141 (16.8)
	More pleasure during sex	145 (17.3)	160 (19.2)	224 (26.7)	169 (20.2)	138 (16.5)
	Better understanding of your sexuality	139 (16.6)	125 (15.1)	235 (28.1)	187 (22.3)	150 (17.9)
	Getting rid of sex related stigmas	151 (18.0)	145 (17.4)	210 (25.0)	179 (21.4)	151 (18.0)
	Improved Self-love and self-compassion	97 (11.6)	148 (17.8)	211 (25.2)	209 (25.0)	171 (20.4)
63	**Part of App**	**Not at all, n (%)**	**Little, n (%)**	**Moderate, n (%)**	**A lot, n (%)**	**A great deal, n (%)**
	Reading/watching articles in the app	92 (11.0)	190 (22.7)	230 (27.6)	188 (22.5)	136 (16.2)
	Chats with Flo's Chatbot	142 (17.0)	160 (29.1)	238 (29.6)	170 (20.3)	126 (15.1)
	Discussion in Secret Chats	186 (22.2)	160 (19.1)	180 (21.6)	161 (19.2)	149 (17.8)
	Courses with Experts	185 (22.1)	167 (20.0)	203 (24.4)	166 (19.8)	115 (13.7)
	Period Predictions	56 (6.7)	110 (13.1)	223 (26.7)	245 (29.3)	202 (24.1)
	Fertile Days and Ovulation Predictions	51 (6.1)	86 (10.3)	212 (25.4)	244 (29.2)	243 (29.0)
	Symptom Tracking	53 (6.3)	134 (16.0)	221 (26.5)	227 (27.1)	201 (24.0)
