# Supplementary material for: Characterization of Self-reported Improvements in Knowledge and Health Among Users of Flo Period Tracking App: Cross-sectional Survey
Source: JMIR Mhealth Uhealth. 2023 Apr 26;11:e40427. doi: 10.2196/40427 (PMC10173043; doi:10.2196/40427)
Supplement: Multimedia Appendix 6 [file mhealth_v11i1e40427_app6.pdf]

Multimedia appendix 6.

Table S1. Age versus reasons for using the app.

**Reason for using the app**	**<34 (n, %)**	**>34 (n, %)**	**Chi-square (*df*)**	***P value***
Menstrual Cycle & ST[a]	1481 (81.9)	313 (77.4)	4.0 (1)	.046
IC[b] and related conditions	404 (22.3)	69 (17.1)	5.1 (1)	.02
Pregnancy Tracking	690 (38.2)	132 (32.7)	4.0 (1)	.04
To help me get pregnant	969 (55.0)	222 (53.6)	0.2 (1)	.66
To help me not get pregnant	214 (11.8)	36 (8.9)	2.5 (1)	.11
Pregnancy loss	115 (6.4)	36 (8.9)	3.0 (1)	.08
Sexual Health	533 (29.6)	92 (22.8)	7.0 (1)	.008
Get tailored health information	627 (34.7)	106 (26.2)	10.2 (1)	.001
To Learn more about my body	759 (42.0)	155 (38.3)	1.6 (1)	.20

[a] Symptom Tracking (ST)

[b] Irregular Cycles (IC)

Table S2. Logistic regression - reasons for using the app versus education (N = 2029).

Why do you use the Flo app? - Menstrual Cycle and symptom tracking

Variable	N (%)	OR (95% CI)	*P*
Education			
Some High School, no Diploma	115 (5.7)	1 (reference)	-
High School Graduate or Diploma	600 (37.4)	1.98 (1.23 - 3.14)	.004
Associate's Degree	139 (6.8)	2.20 (1.18 - 4.17)	.01
Bachelor's Degree	759 (37.4)	1.59 (1.00 - 2.48)	.04
Master's Degree	373 (18.4)	1.39 (0.84 - 2.21)	.19
Doctorate Degree	43 (2.1)	1.22 (0.55 - 2.87)	.64

Why do you use the Flo app? - Irregular cycles and related conditions

Variable	N (%)	OR (95% CI)	*P*
Education			
Some High School, no Diploma	115 (5.7)	1 (reference)	-
High School Graduate or Diploma	600 (37.4)	0.95 (0.60 - 1.51)	.81
Associate's Degree	139 (6.8)	1.16 (0.66 - 2.03)	.61
Bachelor's Degree	759 (37.4)	0.71 (0.45 - 1.14)	.14
Master's Degree	373 (18.4)	0.62 (0.38 - 1.03)	.06
Doctorate Degree	43 (2.1)	0.58 (0.22 - 1.37)	.24

Why do you use the Flo app? - Pregnancy tracking

Variable	N (%)	OR (95% CI)	*P*
Education			
Some High School, no Diploma	115 (5.7)	1 (reference)	-
High School Graduate or Diploma	600 (37.4)	1.17 (0.76 - 1.83)	.48
Associate's Degree	139 (6.8)	1.68 (0.99 - 2.86)	.05
Bachelor's Degree	759 (37.4)	1.79 (1.18 - 2.80)	.007
Master's Degree	373 (18.4)	1.80 (1.15 - 2.86)	.01
Doctorate Degree	43 (2.1)	1.08 (0.49 - 2.29)	.85

Why do you use the Flo app? - Help me get pregnant

Variable	N (%)	OR (95% CI)	*P*
Education			
Some High School, no Diploma	115 (5.7)	1 (reference)	-
High School Graduate or Diploma	600 (37.4)	1.24 (0.83 - 1.85)	.29
Associate's Degree	139 (6.8)	0.92 (0.66 - 1.76)	.77
Bachelor's Degree	759 (37.4)	1.21 (0.82 - 1.80)	.34
Master's Degree	373 (18.4)	1.89 (1.24 - 2.88)	.003
Doctorate Degree	43 (2.1)	1.14 (0.57 - 2.32)	.71

Why do you use the Flo app? - Help me not get pregnant

Variable	N (%)	OR (95% CI)	*P*
Education			
Some High School, no Diploma	115 (5.7)	1 (reference)	-
High School Graduate or Diploma	600 (37.4)	1.71 (0.90 - 3.61)	.13
Associate's Degree	139 (6.8)	1.37 (0.60 - 2.34)	.46
Bachelor's Degree	759 (37.4)	1.20 (0.63 - 2.54)	.60
Master's Degree	373 (18.4)	1.15 (0.57 - 2.52)	.70
Doctorate Degree	43 (2.1)	0.79 (0.17 - 3.61)	.73

Why do you use the Flo app? - Pregnancy loss

Variable	N (%)	OR (95% CI)	*P*
Education			
Some High School, no Diploma	115 (5.7)	1 (reference)	-
High School Graduate or Diploma	600 (37.4)	1.62 (0.73 - 4.30)	.28
Associate's Degree	139 (6.8)	2.36 (0.94 - 6.78)	.08
Bachelor's Degree	759 (37.4)	1.14 (0.51 - 3.05)	.76
Master's Degree	373 (18.4)	1.30 (0.55 - 3.58)	.57
Doctorate Degree	43 (2.1)	1.86 (0.46 - 6.87)	.35

Why do you use the Flo app? - Sexual health

Variable	N (%)	OR (95% CI)	*P*
Education			
Some High School, no Diploma	115 (5.7)	1 (reference)	-
High School Graduate or Diploma	600 (37.4)	0.99 (0.65 - 1.53)	.97
Associate's Degree	139 (6.8)	1.08 (0.64 - 1.82)	.78
Bachelor's Degree	759 (37.4)	0.83 (0.55 - 1.28)	.39
Master's Degree	373 (18.4)	0.61 (0.39 - 0.97)	.04
Doctorate Degree	43 (2.1)	0.48 (0.19 - 1.10)	.10

Why do you use the Flo app? - Get tailored health information

Variable	N (%)	OR (95% CI)	*P*
Education			
Some High School, no Diploma	115 (5.7)	1 (reference)	-

Variable	N (%)	OR (95% CI)	*P*
High School Graduate or Diploma	600 (37.4)	1.18 (0.77 - 1.85)	.45
Associate's Degree	139 (6.8)	1.03 (0.60 - 1.78)	.91
Bachelor's Degree	759 (37.4)	1.35 (0.89 - 2.10)	.17
Master's Degree	373 (18.4)	0.90 (0.57 - 1.43)	.63
Doctorate Degree	43 (2.1)	1.15 (0.53 - 2.42)	.72

Why do you use the Flo app? - Learn more about my body

Variable	N (%)	OR (95% CI)	*P*
Education			
Some High School, no Diploma	115 (5.7)	1 (reference)	-
High School Graduate or Diploma	600 (37.4)	0.98 (0.65 - 1.46)	.91
Associate's Degree	139 (6.8)	0.91 (0.56 - 1.50)	.73
Bachelor's Degree	759 (37.4)	0.81 (0.54 - 1.20)	.28
Master's Degree	373 (18.4)	0.59 (0.39 - 0.91)	.02
Doctorate Degree	43 (2.1)	0.45 (0.21 - 0.95)	.04

Table S3. Logistic regression - reasons for using the app versus age (N = 2212).

Why do you use the Flo app? - Menstrual Cycle and symptom tracking

Variable	N (%)	OR (95% CI)	*P*
Age			
18-24	544 (24.6)	1.92 (1.44 - 2.59)	<.001
25-34	1264 (57.1)	1 (reference)	-
35-44	370 (16.7)	0.89 (0.67 - 1.18)	.41
45-54	34 (1.5)	1.01 (0.46 - 2.53)	.99

Why do you use the Flo app? - Irregular cycles and related conditions

Variable	N (%)	OR (95% CI)	*P*
Age			
18-24	544 (24.6)	1.56 (1.24 - 1.97)	<.001
25-34	1264 (57.1)	1 (reference)	-
35-44	370 (16.7)	0.81 (0.59 - 1.09)	.18
45-54	34 (1.5)	1.04 (0.41 - 2.30)	.92

Why do you use the Flo app? - Pregnancy tracking

Variable	N (%)	OR (95% CI)	*P*
Age			
18-24	544 (24.6)	0.38 (0.30 - 0.48)	<.001
25-34	1264 (57.1)	1 (reference)	-
35-44	370 (16.7)	0.67 (0.53 - 0.86)	<.001
45-54	34 (1.5)	0.08 (0.01 - 0.26)	<.001

Why do you use the Flo app? - Help me get pregnant

Variable	N (%)	OR (95% CI)	*P*
Age			
18-24	544 (24.6)	0.28 (0.23 - 0.35)	<.001
25-34	1264 (57.1)	1 (reference)	-
35-44	370 (16.7)	0.84 (0.67 - 1.07)	.16
45-54	34 (1.5)	0.10 (0.03 - 0.24)	<.001

Why do you use the Flo app? - Help me not get pregnant

Variable	N (%)	OR (95% CI)	*P*
Age			
18-24	544 (24.6)	2.25 (1.69 - 3.01)	<.001
25-34	1264 (57.1)	1 (reference)	-
35-44	370 (16.7)	1.05 (0.69 - 1.54)	.83
45-54	34 (1.5)	0.30 (0.02 - 1.43)	.24

Why do you use the Flo app? - Pregnancy loss

Variable	N (%)	OR (95% CI)	*P*
Age			
18-24	544 (24.6)	0.73 (0.46 - 1.11)	.15
25-34	1264 (57.1)	1 (reference)	-
35-44	370 (16.7)	1.40 (0.92 - 2.09)	.11
45-54	34 (1.5)	0.41 (0.02 - 1.92)	.38

Why do you use the Flo app? - Sexual health

Variable	N (%)	OR (95% CI)	*P*
Age			
18-24	544 (24.6)	1.84 (1.49 - 2.28)	<.001
25-34	1264 (57.1)	1 (reference)	-
35-44	370 (16.7)	0.85 (0.65 - 1.12)	.26
45-54	34 (1.5)	0.90 (0.38 - 1.91)	.79

Why do you use the Flo app? - Get tailored health information

Variable	N (%)	OR (95% CI)	*P*
Age			
18-24	544 (24.6)	2.11 (1.72 - 2.60)	<.001
25-34	1264 (57.1)	1 (reference)	-
35-44	370 (16.7)	0.89 (0.68 - 1.15)	.36
45-54	34 (1.5)	0.51 (0.19 - 1.17)	.14

Why do you use the Flo app? - Learn more about my body

Variable	N (%)	OR (95% CI)	*P*
Age			
18-24	544 (24.6)	2.13 (1.74 - 2.61)	<.001
25-34	1264 (57.1)	1 (reference)	-
35-44	370 (16.7)	1.10 (0.87 - 1.39)	.43
45-54	34 (1.5)	0.95 (0.45 - 1.91)	.89
