# Supplementary material for: Characterization of Self-reported Improvements in Knowledge and Health Among Users of Flo Period Tracking App: Cross-sectional Survey
Source: JMIR Mhealth Uhealth. 2023 Apr 26;11:e40427. doi: 10.2196/40427 (PMC10173043; doi:10.2196/40427)
Supplement: Multimedia Appendix 7 [file mhealth_v11i1e40427_app7.pdf]

Multimedia appendix 7.

Table S1. Education level versus cycle health questions.

Question	Cycle health	Higher education, n (%)	Lower education, n (%)	Chi-square (*df*)	*P value*
16	Overall, did Flo improve your menstrual symptoms (Yes)	610 (77.2)	660 (77.5)	0.0 (1)	.92
17	Flo improved my ovulation symptoms	352 (54.1)	316 (52.2)	0.4 (1)	.55
17	Flo improved my bad mood symptoms	309 (47.4)	322 (59.9)	6.6 (1)	.01
17	Flo improved my cramp symptoms	301 (46.2)	320 (52.9)	5.3 (1)	.02
21	Did Flo help manage your condition and symptoms? (Yes)	143 (65.3)	166 (64.8)	0.0 (1)	.99
25	Did Flo help you seek a HCP for your condition (Yes)	111 (50.7)	108 (42.4)	3.0 (1)	.09
46	Improvement in cycle health education	647 (90.1)	645 (87.9)	1.6 (1)	.20
47	Know if my cycle is regular/irregular	532 (82.2)	553 (85.7)	2.7 (1)	.10
	Know if my cycle is normal/abnormal	490 (75.7)	488 (75.7)	0.0 (1)	.98
	Know if my period flow is normal/abnormal	320 (49.5)	331 (51.3)	0.4 (1)	.54
	Know if I am/am not ovulating	351 (54.3)	362 (56.1)	0.4 (1)	.53
	Know if its normal to have certain symptoms throughout cycle	386 (59.7)	369 (57.2)	0.7 (1)	.40
	Helped me identify whether my discharge was healthy	291 (45.0)	305 (47.3)	0.6 (1)	.44
	Encouraged me to see a doctor, and been diagnosed	49 (7.6)	45 (7.0)	0.1 (1)	.76
	Helped me identify issues related to PCOS	49 (7.6)	62 (9.6)	1.5 (1)	.23
	Helped me identify issues related to endometriosis	18 (2.8)	40 (6.2)	8.0 (1)	.004
	Helped me identify issues related to perimenopause	13 (2.0)	20 (3.1)	1.1 (1)	.29
	Helped me decrease negative effects of PMS	130 (20.0)	126 (19.5)	0.0 (1)	.86
	Helped me communicate with my partner about my cycle	204 (31.5)	198 (30.7)	0.0 (1)	.79
51	How to manage your menstrual cycle symptoms	282 (43.6)	321 (49.8)	4.7 (1)	.03
	How your cycle affects your mental health	383 (59.2)	382 (59.2)	0.0 (1)	.99
	How your cycle affects your physical health	388 (60.0)	388 (60.2)	0.0 (1)	.99
	How your cycle affects your reactions to situations	277 (42.8)	295 (45.7)	1.0 (1)	.32
	How to use your cycle to know when you are most fertile	404 (62.4)	376 (58.3)	2.2 (1)	.14
	How to optimize life around cycle	262 (40.5)	263 (40.8)	0.0 (1)	.96

Table S2. Education level versus general health questions.

Question	General health[a]	Higher education, n (%)	Lower education, n (%)	Chi-square (*df*)	*P value*
53	Sexual Health	408 (34.7)	427 (41.2)	9.5 (1)	.002
	Physical Health	331 (28.2)	258 (24.9)	2.9 (1)	.09
	Mental Health	467 (39.8)	376 (36.3)	2.7 (1)	.10
	Communication with HCP	322 (27.5)	265 (25.6)	0.9 (1)	.35
	Health Behaviours	353 (30.1)	268 (25.8)	4.6 (1)	.03
55	Mental Health				
	Reducing stress	238 (51.0)	188 (50.0)	0.0 (1)	.83
	Reducing anxiety and worry	228 (48.9)	186 (49.5)	0.0 (1)	.91
	Managing Low mood and depressive symptoms	202 (43.4)	183 (48.7)	2.2 (1)	.14
	Improving ability to control emotions	199 (42.6)	170 (45.2)	0.5 (1)	.49
	Being happier	209 (44.8)	185 (49.2)	1.5 (1)	.22
	Having better emotional well-being	239 (51.1)	183 (48.7)	0.4 (1)	.51
	Improving self-esteem	202 (43.3)	180 (47.9)	1.6 (1)	.20
	Improving confidence	202 (43.3)	183 (48.6)	2.2 (1)	.13
	Improving relationships with others	181 (38.8)	170 (45.2)	3.3 (1)	.07
57	Physical Health				
	Feeling less fatigued and tired	225 (68.0)	190 (73.6)	2.0 (1)	.16
	Having more energy	224 (67.7)	187 (72.4)	1.4 (1)	.24
	Sleeping better	221 (66.7)	185 (71.7)	1.4 (1)	.23
	Managing your weight	180 (54.4)	156 (60.5)	2.0 (1)	.16
	Improving your skin	181 (54.6)	165 (64.0)	4.8 (1)	.03
	Improving your fitness	183 (55.3)	168 (65.1)	5.4 (1)	.02
59	Health Behaviour				
	Increased activity/exercise	207 (58.6)	166 (61.9)	0.6 (1)	.45
	Improved diet	226 (64.0)	186 (69.4)	1.7 (1)	.19
	Better stress management	223 (63.2)	189 (70.5)	3.4 (1)	.07
	Reducing harmful habits	194 (55.0)	185 (69.0)	12.1 (1)	<.001
	Better management of symptoms	262 (74.5)	213 (79.5)	1.8 (1)	.17
61	Sexual Health				
	STI's and how to avoid them	199 (48.7)	263 (61.4)	13.1 (1)	<.001
	The signs and symptoms of STI's	185 (45.3)	260 (60.7)	19.3 (1)	<.001
	Safe Sex	248 (60.8)	309 (72.2)	11.7 (1)	<.001
	Contraception Options	236 (57.8)	296 (69.2)	11.1 (1)	<.001
	The signs and symptoms during sex that are indicative of a health issue	250 (61.3)	309 (72.2)	10.6 (1)	<.001
62	Improved Sex Life	291 (71.3)	317 (74.2)	0.8 (1)	.39
	More pleasure during sex	241 (59.0)	290 (67.9)	6.7 (1)	.009
	Better Understanding of your sexuality	270 (66.2)	302 (70.7)	1.8 (1)	.18
	Getting rid of sex related stigmas	253 (62.0)	287 (67.2)	2.2 (1)	.13
	Improved self love and self compassion	286 (70.0)	305 (71.4)	0.1 (1)	.72
64	Communication with HCP				
	Asking questions about my health/body	223 (69.3)	187 (70.6)	0.0 (1)	.80
	Asking for resources I think I need	137 (42.5)	137 (51.7)	4.5 (1)	.03
	Sharing with what's going on in my body	256 (79.5)	195 (73.6)	2.5 (1)	.11
	Understanding of my reproductive health	237 (73.6)	187 (70.6)	0.5 (1)	.47

[a]All general health questions were compared for participant responses from ‘a moderate to a great deal’.

Table S3. Education level versus pregnancy questions.

Question	Pregnancy	Higher education, n (%)	Lower education, n (%)	Chi-square (*df*)	*P value*
29	Did you get pregnant whilst using Flo? (Yes)	398 (81.3)	229 (68.4)	17.8 (1)	<.001
30	Did Flo help you get pregnant? (Yes)	290 (72.8)	171 (74.7)	0.2 (1)	.69
32	Did Flo help you prepare for a healthy pregnancy? (Yes)	338 (84.9)	192 (83.8)	0.1 (1)	.81
33	Did Flo help you prepare for a natural labour? (Yes)	28 (30.4)	42 (39.6)	1.3 (1)	.25
34	Improvement in Pregnancy Education	427 (87.5)	271 (80.9)	6.2 (1)	.01
36	Know more about my baby development	357 (83.8)	220 (81.2)	0.6 (1)	.42
	Know more about my body during pregnancy	372 (87.3)	217 (80.1)	6.1 (1)	.01
	Know more about the basic do's and don'ts	306 (71.9)	186 (68.6)	0.7 (1)	.41
	Know more about what symptoms are OK and not during pregnancy	309 (72.6)	196 (72.3)	0.0 (1)	.99
	Know more about diet and nutrition	236 (55.5)	159 (58.7)	0.6 (1)	.44
	Know more about prenatal care	225 (52.9)	154 (56.8)	0.9 (1)	.34
39	How to manage your pregnancy symptoms	238 (55.9)	165 (60.9)	1.5 (1)	.22
	How pregnancy affects mental health	229 (53.8)	155 (57.2)	0.6 (1)	.42
	How pregnancy affects physical health	328 (77.0)	181 (66.8)	8.2 (1)	.004
	How pregnancy affects reaction to situations	149 (35.0)	108 (39.9)	1.5 (1)	.22
	How to optimize life around pregnancy	251 (58.8)	142 (52.4)	2.6 (1)	.11
	How to manage postpartum symptoms	77 (18.1)	72 (26.6)	6.6 (1)	.01
	How to reduce risk for preterm labour	91 (21.4)	76 (28.0)	3.7 (1)	.05
