# Supplementary material for: Characterization of Self-reported Improvements in Knowledge and Health Among Users of Flo Period Tracking App: Cross-sectional Survey
Source: JMIR Mhealth Uhealth. 2023 Apr 26;11:e40427. doi: 10.2196/40427 (PMC10173043; doi:10.2196/40427)
Supplement: Multimedia Appendix 8 [file mhealth_v11i1e40427_app8.pdf]

Multimedia appendix 8.

Table S1. High-income and low- and middle-income countries versus pregnancy questions.

Question	Pregnancy	HIC, n (%)	LMIC, n (%)	Chi-square (*df*)	*P value*
29	Did you get pregnant whilst using Flo? (Yes)	489 (79.8)	138 (65.4)	17.0 (1)	<.001
30	Did Flo help you get pregnant? (Yes)	354 (72.3)	107 (77.5)	1.2 (1)	.27
32	Did Flo help you prepare for a healthy pregnancy? (Yes)	409 (83.6)	121 (87.7)	1.1 (1)	.30
33	Did Flo help you prepare for natural labour? (Yes)	39 (31.5)	31 (42.5)	2.0 (1)	.16
34	Improvement in Pregnancy Education	525 (85.8)	173 (82.0)	1.5 (1)	.23
36	Know more about my baby development	444 (86.5)	133 (79.6)	4.2 (1)	.04
	Know more about my body during pregnancy	447 (87.1)	142 (85.0)	0.6 (1)	.57
	Know more about the basic do's and don'ts	369 (71.9)	123 (73.7)	0.1 (1)	.74
	Know more about what symptoms are OK and not during pregnancy	381 (74.3)	124 (74.3)	0.0 (1)	.99
	Know more about diet and nutrition	306 (59.7)	89 (53.3)	1.8 (1)	.18
	Know more about prenatal care	289 (56.3)	90 (53.9)	0.2 (1)	.64
39	How to manage your pregnancy symptoms	293 (61.9)	110 (68.8)	2.1 (1)	.15
	How pregnancy affects mental health	281 (59.4)	103 (64.4)	1.0 (1)	.31
	How pregnancy affects physical health	387 (81.8)	122 (76.3)	2.0 (1)	.16
	How pregnancy affects reaction to situations	189 (40.0)	68 (42.5)	0.2 (1)	.64
	How to optimize life around pregnancy	289 (62.0)	100 (62.5)	0.0 (1)	.98
	How to manage postpartum symptoms	97 (20.5)	52 (32.5)	8.9 (1)	.002
	How to reduce risk for preterm labour	109 (23.0)	58 (36.3)	10.1 (1)	.002

[a] High-income country (HIC), low- and middle-income country (LMIC).

Table S2. High-income and low- and middle-income countries versus general health questions.

Question	General health[a]	HIC[b], n (%)	LMIC[b],n (%)	Chi-square (*df*)	*P value*
53	Sexual Health	526 (34.8)	309 (44.2)	17.7 (1)	<.001
	Physical Health	354 (23.4)	235 (33.6)	25.1 (1)	<.001
	Mental Health	553 (36.5)	290 (41.5)	4.7 (1)	.03
	Communication with HCP	426 (28.2)	161 (23.0)	6.2 (1)	.01
	Health Behaviours	407 (26.9)	214 (30.6)	3.1 (1)	.08
55	Mental Health				
	Reducing stress	280 (50.6)	146 (50.3)	0.0 (1)	.99
	Reducing anxiety and worry	275 (49.7)	139 (47.9)	0.2 (1)	.67
	Managing Low mood and depressive symptoms	258 (46.6)	127 (43.8)	0.5 (1)	.47
	Improving ability to control emotions	239 (43.2)	130 (44.8)	0.1 (1)	.71
	Being happier	261 (47.2)	133 (45.9)	0.1 (1)	.77
	Having better emotional well-being	274 (49.5)	148 (51.0)	0.1 (1)	.74
	Improving self-esteem	239 (43.2)	143 (49.3)	2.6 (1)	.10
	Improving confidence	248 (44.8)	137 (47.2)	0.3 (1)	.55
	Improving relationships with others	218 (39.4)	133 (45.9)	3.0 (1)	.08
57	Physical Health				
	Feeling less fatigued and tired	240 (67.8)	175 (74.5)	2.7 (1)	.09
	Having more energy	235 (66.4)	176 (74.9)	4.5 (1)	.03
	Sleeping better	230 (65.0)	176 (74.9)	6.0 (1)	.01
	Managing your weight	195 (55.0)	141 (60.0)	1.2 (1)	.27
	Improving your skin	200 (56.5)	146 (62.1)	1.6 (1)	.20
	Improving your fitness	201 (56.8)	150 (63.8)	2.6 (1)	.10
59	Health Behaviour				
	Increased activity/exercise	231 (56.8)	142 (66.4)	5.0 (1)	.03
	Improved diet	263 (64.6)	149 (69.6)	1.4 (1)	.24
	Better stress management	257 (63.1)	155 (72.4)	5.0 (1)	.03
	Reducing harmful habits	235 (57.7)	144 (67.3)	5.0 (1)	.03
	Better management of symptoms	301 (74.0)	175 (81.8)	4.6 (1)	.04
61	Sexual Health				
	STI's and how to avoid them	271 (51.4)	191 (61.8)	8.1 (1)	.004
	The signs and symptoms of STI's	269 (51.0)	176 (57.0)	2.5 (1)	.11
	Safe Sex	323 (61.3)	234 (75.7)	17.6 (1)	<.001
	Contraception Options	328 (62.2)	204 (66.0)	1.0 (1)	.31
	The signs and symptoms during sex that are indicative of a health issue	345 (65.5)	214 (69.3)	1.1 (1)	.29
62	Improved Sex Life	371 (70.5)	237 (76.7)	3.4 (1)	.06
	More pleasure during sex	319 (60.6)	212 (68.6)	5.0 (1)	.03
	Better Understanding of your sexuality	335 (63.7)	237 (76.7)	14.7 (1)	<.001
	Getting rid of sex related stigmas	333 (63.3)	207 (67.0)	1.0 (1)	.32
	Improved self love and self compassion	364 (69.2)	227 (73.5)	1.5 (1)	.22
64	Communication with HCP				
	Asking questions about my health/body	290 (69.4)	120 (75.0)	1.5 (1)	.22
	Asking for resources I think I need	205 (49.0)	69 (43.1)	1.4 (1)	.24
	Sharing with what's going on in my body	335 (80.1)	116 (72.5)	3.5 (1)	.06
	Understanding of my reproductive health	308 (73.4)	72.5 (72.5)	0.0 (1)	.85

[a]All general health questions were compared for participant responses from ‘a moderate to a great deal’.

[b] High-income country (HIC), low- and middle-income country (LMIC).

Table S3. High-income and low- and middle-income countries versus cycle health questions.

Question	Cycle health	HIC, n (%)	LMIC, n (%)	Chi-square (*df*)	*P value*
16	Overall, did Flo improve your menstrual symptoms (Yes)	848 (81.9)	422 (94.1)	24.4 (1)	<.001
17	Flo improved my ovulation symptoms	450 (53.8)	218 (52.0)	0.3 (1)	.56
17	Flo improved my bad mood symptoms	399 (47.7)	222 (52.9)	2.7 (1)	.09
17	Flo improved my cramp symptoms	438 (52.4)	203 (48.3)	1.8 (1)	.18
21	Did Flo help manage your condition and symptoms? (Yes)	210 (77.7)	99 (86.4)	0.1 (1)	.84
25	Did Flo help you seek a HCP for your condition (Yes)	131 (40.3)	88 (59.1)	13.7 (1)	<.001
46	Improvement in Cycle Health Education	838 (88.0)	454 (90.1)	2.3 (1)	.13
47	Know if my cycle is regular/irregular	698 (83.5)	387 (85.4)	0.6 (1)	.43
	Know if my cycle is normal/abnormal	629 (75.3)	349 (77.0)	0.4 (1)	.54
	Know if my period flow is normal/abnormal	400 (47.9)	251 (55.4)	6.3 (1)	.01
	Know if I am/am not ovulating	466 (55.9)	247 (54.5)	0.1 (1)	.70
	Know if its normal to have certain symptoms throughout cycle	499 (59.8)	256 (56.5)	1.5 (1)	.28
	Helped me identify whether my discharge was healthy	382 (45.7)	214 (47.2)	0.2 (1)	.65
	Encouraged me to see a doctor, and been diagnosed	43 (5.1)	51 (11.3)	15.3 (1)	<.001
	Helped me identify issues related to PCOS	59 (7.1)	52 (11.5)	6.7 (1)	.001
	Helped me identify issues related to endometriosis	39 (4.5)	19 (4.2)	0.1 (1)	.80
	Helped me identify issues related to perimenopause	17 (2.0)	16 (3.5)	2.1 (1)	.15
	Helped me decrease negative effects of PMS	157 (18.8)	99 (21.9)	1.5 (1)	.22
	Helped me communicate with my partner about my cycle	280 (33.5)	122 (26.9)	5.7 (1)	.02
51	How to manage your menstrual cycle symptoms	347 (43.6)	256 (58.3)	23.8 (1)	<.001
	How your cycle affects your mental health	503 (63.3)	262 (60.0)	1.4 (1)	.24
	How your cycle affects your physical health	511 (64.3)	265 (60.4)	1.7 (1)	.19
	How your cycle affects your reactions to situations	383 (48.2)	189 (43.1)	2.8 (1)	.10
	How to use your cycle to know when you are most fertile	515 (64.8)	265 (60.4)	2.2 (1)	.14
	How to optimize life around cycle	324 (40.8)	201 (45.8)	2.7 (1)	.10

[a] High-income country (HIC), low- and middle-income country (LMIC).
