# Supplementary material for: Characterization of Self-reported Improvements in Knowledge and Health Among Users of Flo Period Tracking App: Cross-sectional Survey
Source: JMIR Mhealth Uhealth. 2023 Apr 26;11:e40427. doi: 10.2196/40427 (PMC10173043; doi:10.2196/40427)
Supplement: Multimedia Appendix 9 [file mhealth_v11i1e40427_app9.pdf]

Multimedia appendix 9.

Table S1: Premium and free users versus pregnancy questions.

Question	Pregnancy	Premium, n (%)	Free, n (%)	Chi-square (*df*)	*P value*
29	Did you get pregnant whilst using Flo? (Yes)	475 (85.6)	93 (48.5)	108.7 (1)	<.001
30	Did Flo help you get pregnant? (Yes)	357 (75.0)	62 (66.7)	2.5 (1)	.12
32	Did Flo help you prepare for a healthy pregnancy? (Yes)	410 (86.3)	65 (77.4)	4.1 (1)	.04
33	Did Flo help you prepare for a natural labour? (Yes)	25 (31.3)	39 (39.0)	0.7 (1)	.39
34	Improvement in Pregnancy Education	501 (90.6)	133 (68.9)	51.0 (1)	<.001
36	Know more about my baby development	434 (88.0)	88 (70.4)	22.3 (1)	<.001
	Know more about my body during pregnancy	437 (88.6)	99 (79.2)	6.9 (1)	.009
	Know more about the basic do's and don'ts	372 (75.5)	76 (60.8)	10.0 (1)	.002
	Know more about what symptoms are OK and not during pregnancy	370 (75.1)	89 (71.2)	0.6 (1)	.44
	Know more about diet and nutrition	304 (61.7)	55 (44.0)	12.2 (1)	<.001
	Know more about prenatal care	285 (57.8)	56 (48.8)	6.3 (1)	.01
39	How to manage your pregnancy symptoms	287 (62.1)	72 (63.7)	0.1 (1)	.84
	How pregnancy affects mental health	284 (61.5)	63 (55.6)	1.0 (1)	.31
	How pregnancy affects physical health	388 (84.0)	72 (63.7)	22.1 (1)	<.001
	How pregnancy affects reaction to situations	183 (39.6)	39 (43.4)	0.4 (1)	.53
	How to optimize life around pregnancy	303 (65.6)	57 (50.4)	8.3 (1)	.004
	How to manage postpartum symptoms	87 (18.8)	38 (33.6)	10.8 (1)	<.001
	How to reduce risk for preterm labor	105 (22.7)	42 (37.2)	9.2 (1)	.002

Table S2. Premium and free users versus cycle health questions.

Question	Cycle health	Premium, n (%)	Free, n (%)	Chi-square (*df*)	*P value*
16	Overall, did Flo improve your menstrual symptoms (Yes)	627 (78.5)	541 (78.3)	0.0 (1)	.95
17	Flo improved my ovulation symptoms	365 (58.8)	248 (46.5)	17.0 (1)	<.001
17	Flo improved my bad mood symptoms	325 (52.3)	272 (50.8)	0.2 (1)	.68
17	Flo improved my cramp symptoms	289 (46.5)	276 (51.6)	2.8 (1)	.09
21	Did Flo help manage your condition and symptoms? (Yes)	145 (66.2)	135 (64.0)	0.1 (1)	.70
25	Did Flo help you seek a HCP for your condition (Yes)	93 (42.5)	105 (50.0)	2.2 (1)	.14
46	Improvement in Cycle Health Education	524 (92.7)	646 (87.3)	9.7 (1)	.002
47	Know if my cycle is regular/irregular	430 (82.2)	555 (86.0)	2.9 (1)	.09
	Know if my cycle is normal/abnormal	401 (76.7)	481 (74.6)	0.6 (1)	.45
	Know if my period flow is normal/abnormal	272 (52.0)	312 (48.4)	1.4 (1)	.24
	Know if I am/am not ovulating	303 (57.9)	356 (55.2)	0.8 (1)	.38
	Know if its normal to have certain symptoms throughout cycle	345 (66.0)	354 (58.9)	14.3 (1)	<.001
	Helped me identify whether my discharge was healthy	276 (52.8)	268 (41.6)	14.2 (1)	<.001
	Encouraged me to see a doctor, and been diagnosed	34 (6.5)	52 (8.1)	0.8 (1)	.37
	Helped me identify issues related to PCOS	45 (8.7)	56 (8.6)	0.0 (1)	.99
	Helped me identify issues related to endometriosis	26 (5.0)	26 (4.0)	0.4 (1)	.53
	Helped me identify issues related to perimenopause	11 (2.1)	16 (2.5)	0.0 (1)	.82
	Helped me decrease negative effects of PMS	117 (22.4)	109 (16.9)	5.2 (1)	.02
	Helped me communicate with my partner about my cycle	209 (40.0)	160 (24.8)	30.0 (1)	<.001
51	How to manage your menstrual cycle symptoms	227 (45.4)	320 (51.8)	4.3 (1)	.04
	How your cycle affects your mental health	321 (64.2)	366 (59.2)	2.7 (1)	.10
	How your cycle affects your physical health	341 (68.2)	360 (58.3)	11.3 (1)	<.001
	How your cycle affects your reactions to situations	245 (49.0)	264 (42.7)	4.1 (1)	.04
	How to use your cycle to know when you are most fertile	343 (68.6)	359 (58.0)	12.6 (1)	<.001
	How to optimize life around cycle	230 (46.0)	256 (41.4)	2.2 (1)	.14

Table S3. Premium and free users versus general health questions.

Question	General health[a]	Premium, n (%)	Free, n (%)	Chi-square (*df*)	*P value*
53	Sexual Health	421 (39.1)	336 (36.8)	1.0 (1)	.31
	Physical Health	313 (29.1)	221 (24.1)	5.7 (1)	.02
	Mental Health	439 (40.7)	321 (35.0)	6.6 (1)	.01
	Communication with HCP	345 (32.2)	183 (20.0)	35.9 (1)	<.001
	Health Behaviours	354 (32.9)	208 (22.7)	24.3 (1)	<.001
55	Mental Health				
	Reducing stress	230 (52.2)	154 (47.9)	1.3 (1)	.26
	Reducing anxiety and worry	218 (49.7)	152 (47.4)	0.3 (1)	.57
	Managing Low mood and depressive symptoms	207 (47.2)	233 (43.0)	1.1 (1)	.29
	Improving ability to control emotions	197 (44.9)	133 (41.4)	0.9 (1)	.38
	Being happier	213 (48.5)	141 (44.0)	1.4 (1)	.24
	Having better emotional well-being	223 (50.8)	154 (48.0)	0.5 (1)	.49
	Improving self-esteem	194 (44.2)	144 (44.9)	0.0 (1)	.91
	Improving confidence	195 (44.34	150 (46.7)	0.3 (1)	.58
	Improving relationships with others	176 (40.1)	137 (42.7)	0.4 (1)	.52
57	Physical Health				
	Feeling less fatigued and tired	220 (70.0)	156 (70.6)	0.0 (1)	.99
	Having more energy	217 (69.1)	156 (70.6)	0.1 (1)	.83
	Sleeping better	217 (69.1)	157 (71.0)	0.1 (1)	.74
	Managing your weight	180 (57.3)	124 (56.1)	0.1 (1)	.82
	Improving your skin	185 (58.9)	130 (58.9)	0.0 (1)	.99
	Improving your fitness	185 (58.9)	132 (59.7)	0.0 (1)	.96
59	Health Behaviour				
	Increased activity/exercise	217 (61.3)	127 (61.1)	0.0 (1)	.99
	Improved diet	242 (68.3)	133 (63.9)	1.0 (1)	.33
	Better stress management	235 (66.4)	140 (67.3)	0.0 (1)	.90
	Reducing harmful habits	213 (60.2)	132 (63.5)	0.5 (1)	.49
	Better management of symptoms	275 (77.7)	158 (76.0)	0.1 (1)	.72
61	Sexual Health				
	STI's and how to avoid them	220 (52.1)	194 (57.6)	2.2 (1)	.14
	The signs and symptoms of STI's	215 (50.9)	180 (53.4)	0.4 (1)	.52
	Safe Sex	274 (64.9)	233 (69.1)	1.5 (1)	.23
	Contraception Options	263 (62.3)	218 (64.7)	0.5 (1)	.42
	The signs and symptoms during sex that are indicative of a health issue	282 (66.8)	221 (65.6)	0.1 (1)	.82
62	Improved Sex Life	312 (74.1)	244 (72.4)	0.1 (1)	.71
	More pleasure during sex	267 (63.4)	218 (64.7)	0.1 (1)	.73
	Better Understanding of your sexuality	284 (67.5)	242 (71.8)	1.6 (1)	.20
	Getting rid of sex related stigmas	280 (66.5)	212 (62.9)	0.8 (1)	.37
	Improved self love and self compassion	304 (72.2)	231 (68.5)	0.9 (1)	.34
64	Communication with HCP				
	Asking questions about my health/body	249 (73.7)	125 (68.7)	1.2 (1)	.27
	Asking for resources I think I need	154 (45.6)	89 (48.9)	0.4 (1)	.52
	Sharing with what's going on in my body	274 (81.1)	133 (73.1)	4.0 (1)	.046
	Understanding of my reproductive health	256 (75.7)	124 (68.1)	3.1 (1)	.08

[a]All general health questions were compared for participant responses from ‘a moderate to a great deal’.
