# Supplementary material for: Characterization of Self-reported Improvements in Knowledge and Health Among Users of Flo Period Tracking App: Cross-sectional Survey
Source: JMIR Mhealth Uhealth. 2023 Apr 26;11:e40427. doi: 10.2196/40427 (PMC10173043; doi:10.2196/40427)
Supplement: Multimedia Appendix 10 [file mhealth_v11i1e40427_app10.pdf]

Multimedia appendix 10.

Table S1. Frequent and infrequent app use versus pregnancy questions.

Question	Pregnancy	Frequent, n (%)	Infrequent, n (%)	Chi-square (*df*)	*P value*
29	Did you get pregnant whilst using Flo? (Yes)	555 (80.2)	72 (54.1)	40.6 (1)	<.001
30	Did Flo help you get pregnant? (Yes)	416 (74.8)	45 (62.5)	4.5 (1)	.03
32	Did Flo help you prepare for a healthy pregnancy? (Yes)	477 (85.8)	54 (75.0)	4.9 (1)	.03
33	Did Flo help you prepare for a natural labour? (Yes)	49 (35.8)	21 (34.4)	<0.1 (1)	.96
34	Improvement in Pregnancy Education	601 (87.0)	97 (72.4)	16.2 (1)	<.001
36	Know more about my baby development	506 (86.3)	71 (75.5)	6.6 (1)	.01
	Know more about my body during pregnancy	516 (88.1)	73 (77.7)	6.7 (1)	.01
	Know more about the basic do's and don'ts	433 (73.9)	59 (62.8)	4.5 (1)	.03
	Know more about what symptoms are OK and not during pregnancy	438 (74.7)	67 (71.3)	0.3 (1)	.56
	Know more about diet and nutrition	346 (59.0)	49 (52.7)	1.3 (1)	.25
	Know more about prenatal care	333 (56.8)	46 (48.9)	1.7 (1)	.19
39	How to manage your pregnancy symptoms	349 (64.0)	54 (61.4)	0.1 (1)	.72
	How pregnancy affects mental health	333 (61.1)	51 (58.0)	0.2 (1)	.66
	How pregnancy affects physical health	446 (81.8)	63 (71.6)	4.4 (1)	.04
	How pregnancy affects reaction to situations	222 (40.7)	35 (40.0)	0.0 (1)	.96
	How to optimize life around pregnancy	342 (62.8)	51 (58.0)	0.6 (1)	.46
	How to manage postpartum symptoms	123 (22.6)	26 (29.5)	1.7 (1)	.19
	How to reduce risk for preterm labour	143 (26.2)	24 (27.3)	0.0 (1)	.94

Table S2. Frequent and infrequent app use versus cycle health questions.

Question	Cycle health	Frequent, n (%)	Infrequent, n (%)	Chi-square (*df*)	*P value*
16	Overall, did Flo improve your Menstrual symptoms (Yes)	958 (78.5)	312 (74.1)	3.2 (1)	.07
17	Flo improved my ovulation symptoms	541 (56.7)	127 (42.2)	19.2 (1)	<.001
17	Flo improved my bad mood symptoms	484 (50.7)	157 (51.8)	0.1 (1)	.75
17	Flo improved my cramp symptoms	654 (48.6)	157 (51.8)	0.9 (1)	.34
21	Did Flo help manage your condition and symptoms? (Yes)	219 (67.6)	90 (59.6)	2.6 (1)	.11
25	Did Flo help you seek a HCP for your condition (Yes)	156 (48.1)	63 (42.0)	1.3 (1)	.25
46	Improvement in Cycle Health Education	907 (89.4)	385 (88.1)	0.4 (1)	.54
47	Know if my cycle is regular/irregular	757 (83.6)	328 (85.6)	0.7 (1)	.42
	Know if my cycle is normal/abnormal	693 (76.6)	285 (74.4)	0.6 (1)	.45
	Know if my period flow is normal/abnormal	463 (51.1)	188 (49.0)	0.4 (1)	.54
	Know if I am/am not ovulating	515 (56.9)	198 (51.7)	2.7 (1)	.10
	Know if its normal to have certain symptoms throughout cycle	551 (60.9)	204 (53.2)	6.1 (1)	.01
	Helped me identify whether my discharge was healthy	432 (47.8)	164 (42.8)	2.4 (1)	.12
	Encouraged me to see a doctor, and been diagnosed	71 (7.8)	23 (6.0)	1.1 (1)	.30
	Helped me identify issues related to PCOS	85 (9.4)	26 (6.8)	2.0 (1)	.16
	Helped me identify issues related to endometriosis	48 (5.3)	10 (2.6)	3.9 (1)	.047
	Helped me identify issues related to perimenopause	21 (2.3)	12 (3.1)	0.4 (1)	.52
	Helped me decrease negative effects of PMS	184 (20.3)	72 (18.8)	0.3 (1)	.58
	Helped me communicate with my partner about my cycle	325 (35.9)	77 (20.1)	30.6 (1)	<.001
51	How to manage your menstrual cycle symptoms	419 (47.9)	184 (51.1)	0.9 (1)	.34
	How your cycle affects your mental health	541 (61.9)	224 (62.2)	0.0 (1)	.97
	How your cycle affects your physical health	558 (63.8)	218 (60.6)	1.0 (1)	.31
	How your cycle affects your reactions to situations	395 (45.2)	177 (49.2)	1.5 (1)	.23
	How to use your cycle to know when you are most fertile	597 (68.3)	183 (50.8)	32.7 (1)	<.001
	How to optimize life around cycle	370 (42.3)	155 (43.1)	0.01 (1)	0.87

Table S3. Frequent and infrequent app use versus general health questions.

Question	General health[a]	Frequent, n (%)	Infrequent, n (%)	Chi-square (*df*)	*P value*
53	Sexual Health	668 (40.4)	167 (29.8)	20.2 (1)	<.001
	Physical Health	445 (28.4)	144 (25.6)	0.3 (1)	.57
	Mental Health	639 (38.7)	204 (36.2)	1.0 (1)	.33
	Communication with HCP	471 (28.5)	116 (20.6)	13.1 (1)	<.001
	Health Behaviours	482 (29.2)	139 (24.7)	3.9 (1)	.047
55	Mental Health				
	Reducing stress	315 (49.3)	111 (54.4)	1.4 (1)	.23
	Reducing anxiety and worry	308 (48.2)	106 (52.0)	0.7 (1)	.39
	Managing Low mood and depressive symptoms	288 (45.1)	97 (47.5)	0.29 (1)	.59
	Improving ability to control emotions	278 (43.5)	91 (44.6)	0.1 (1)	.85
	Being happier	299 (46.8)	95 (46.6)	0.0 (1)	.99
	Having better emotional well-being	319 (49.9)	103 (50.5)	0.0 (1)	.95
	Improving self-esteem	281 (44.0)	101 (49.5)	1.7 (1)	.19
	Improving confidence	284 (44.4)	101 (59.5)	1.4 (1)	.24
	Improving relationships with others	254 (39.7)	97 (47.5)	3.6 (1)	.06
57	Physical Health				
	Feeling less fatigued and tired	312 (70.0)	103 (71.5)	0.1 (1)	.83
	Having more energy	306 (68.8)	105 (72.9)	0.7 (1)	.40
	Sleeping better	299 (67.2)	107 (74.3)	2.2 (1)	.13
	Managing your weight	253 (56.9)	83 (57.6)	0.0 (1)	.95
	Improving your skin	261 (58.7)	85 (59.0)	0.0 (1)	.99
	Improving your fitness	266 (59.8)	85 (59.0)	0.0 (1)	.95
59	Health Behaviour				
	Increased activity/exercise	291 (60.4)	82 (58.9)	0.1 (1)	.85
	Improved diet	326 (67.6)	86 (61.9)	1.4 (1)	.24
	Better stress management	317 (65.7)	95 (68.3)	0.2 (1)	.64
	Reducing harmful habits	295 (61.1)	84 (60.4)	0.0 (1)	.95
	Better management of symptoms	371 (77.0)	105 (75.5)	0.1 (1)	.81
61	Sexual Health				
	STI's and how to avoid them	374 (55.9)	88 (52.3)	0.5 (1)	.51
	The signs and symptoms of STI's	360 (53.8)	85 (50.9)	0.4 (1)	.56
	Safe Sex	446 (66.7)	111 (66.5)	0.0 (1)	.99
	Contraception Options	427 (63.8)	105 (62.9)	0.0 (1)	.89
	The signs and symptoms during sex that are indicative of a health issue	447 (66.8)	112 (67.1)	0.0 (1)	.99
62	Improved Sex Life	492 (73.6)	116 (69.5)	1.0 (1)	.32
	More pleasure during sex	426 (63.8)	105 (62.5)	0.0 (1)	.90
	Better Understanding of your sexuality	460 (68.9)	112 (66.7)	0.1 (1)	.72
	Getting rid of sex related stigmas	428 (64.1)	112 (67.1)	0.4 (1)	.53
	Improved self love and self compassion	473 (70.8)	118 (70.6)	0.0 (1)	.99
64	Communication with HCP				
	Asking questions about my health/body	342 (73.3)	68 (60.7)	6.4 (1)	.01
	Asking for resources I think I need	226 (48.5)	38 (42.9)	0.9 (1)	.33
	Sharing with what's going on in my body	366 (78.5)	85 (75.9)	0.2 (1)	.63
	Understanding of my reproductive health	355 (76.2)	69 (61.6)	9.1 (1)	.003

[a]All general health questions were compared for participant responses from ‘a moderate to a great deal’.
