# Supplementary material for: Characterization of Self-reported Improvements in Knowledge and Health Among Users of Flo Period Tracking App: Cross-sectional Survey
Source: JMIR Mhealth Uhealth. 2023 Apr 26;11:e40427. doi: 10.2196/40427 (PMC10173043; doi:10.2196/40427)
Supplement: Multimedia Appendix 11 [file mhealth_v11i1e40427_app11.pdf]

Multimedia appendix 11.

Table S1. Long- and short-term app use versus cycle health questions.

Question	Cycle health	Long-term, n (%)	Short-term, n (%)	Chi-square (*df*)	*P value*
16	Overall, did Flo improve your menstrual symptoms (Yes)	821 (77.8)	449 (76.6)	0.2 (1)	.62
17	Flo improved my ovulation symptoms	424 (52.1)	244 (55.3)	1.1 (1)	.29
17	Flo improved my bad mood symptoms	430 (52.7)	211 (47.8)	2.6 (1)	.11
17	Flo improved my cramp symptoms	414 (50.7)	207 (46.9)	1.6 (1)	.21
21	Did Flo help manage your condition and symptoms? (Yes)	213 (68.1)	96 (63.2)	3.3 (1)	.07
25	Did Flo help you seek a HCP for your condition (Yes)	153 (48.9)	66 (41.0)	2.4 (1)	.13
46	Improvement in Cycle Health Education	750 (89.4)	542 (88.4)	0.3 (1)	.62
47	Know if my cycle is regular/irregular	651 (87.0)	434 (80.4)	10.0 (1)	.002
	Know if my cycle is normal/abnormal	586 (78.3)	392 (72.6)	5.4 (1)	.02
	Know if my period flow is normal/abnormal	407 (54.4)	244 (45.2)	10.3 (1)	.001
	Know if I am/am not ovulating	427 (57.1)	286 (53.0)	2.0 (1)	.16
	Know if its normal to have certain symptoms throughout cycle	465 (62.2)	290 (53.7)	8.9 (1)	.003
	Helped me identify whether my discharge was healthy	326 (48.4)	234 (43.3)	3.0 (1)	.08
	Encouraged me to see a doctor, and been diagnosed	63 (8.4)	31 (5.7)	2.9 (1)	.09
	Helped me identify issues related to PCOS	78 (10.4)	33 (6.1)	6.9 (1)	.009
	Helped me identify issues related to endometriosis	40 (5.3)	18 (3.3)	2.5 (1)	.11
	Helped me identify issues related to perimenopause	21 (2.8)	12 (2.2)	0.2 (1)	.63
	Helped me decrease negative effects of PMS	176 (23.5)	80 (14.8)	14.4 (1)	<.001
	Helped me communicate with my partner about my cycle	218 (29.1)	184 (34.1)	3.3 (1)	.07
51	How to manage your menstrual cycle symptoms	374 (51.9)	229 (44.6)	6.0 (1)	.01
	How your cycle affects your mental health	477 (66.2)	288 (56.1)	12.3 (1)	<.001
	How your cycle affects your physical health	486 (67.4)	290 (56.5)	14.7 (1)	<.001
	How your cycle affects your reactions to situations	361 (50.0)	211 (41.1)	9.3 (1)	.002
	How to use your cycle to know when you are most fertile	433 (60.0)	347 (67.4)	8.1 (1)	.008
	How to optimize life around cycle	326 (45.2)	199 (38.8)	4.8 (1)	.03

Table S2. Long- and short-term app use versus pregnancy questions.

Question	Pregnancy	Long-term, n (%)	Short-term, n (%)	Chi-square (*df*)	*P value*
29	Did you get pregnant whilst using Flo? (Yes)	405 (81.0)	222 (68.5)	16.2 (1)	<.001
30	Did Flo help you get pregnant? (Yes)	287 (70.9)	174 (78.4)	3.8 (1)	.05
32	Did Flo help you prepare for a healthy pregnancy? (Yes)	340 (84.0)	190 (85.6)	0.2 (1)	.67
33	Did Flo help you prepare for a natural labour? (Yes)	32 (33.7)	38 (37.3)	0.1 (1)	.71
34	Improvement in Pregnancy Education	426 (85.4)	272 (84.0)	0.2 (1)	.65
36	Know more about my baby development	367 (87.4)	210 (80.8)	5.0 (1)	.03
	Know more about my body during pregnancy	368 (87.6)	221 (85.0)	0.7 (1)	.39
	Know more about the basic do's and don'ts	297 (70.7)	195 (75.0)	1.3 (1)	.26
	Know more about what symptoms are OK and not during pregnancy	324 (77.1)	181 (69.6)	4.4 (1)	.04
	Know more about diet and nutrition	248 (59.0)	147 (56.5)	0.3 (1)	.57
	Know more about prenatal care	241 (57.4)	138 (53.1)	1.0 (1)	.31
39	How to manage your pregnancy symptoms	268 (68.5)	135 (55.8)	10.0 (1)	.002
	How pregnancy affects mental health	248 (63.4)	136 (56.2)	3.0 (1)	.08
	How pregnancy affects physical health	318 (81.3)	191 (78.9)	0.4 (1)	.52
	How pregnancy affects reaction to situations	171 (43.7)	86 (35.5)	3.8 (1)	.05
	How to optimize life around pregnancy	252 (64.5)	141 (58.3)	2.2 (1)	.14
	How to manage postpartum symptoms	119 (30.4)	30 (12.4)	26.0 (1)	<.001
	How to reduce risk for preterm labour	117 (29.9)	50 (20.7)	6.1 (1)	.01

Table S3. Long- and short-term app use versus general health questions.

Question	General health	Long-term, n (%)	Short-term, n (%)	Chi-square (*df*)	*P value*
53	Sexual Health	533 (40.9)	304 (33.5)	11.7 (1)	<.001
	Physical Health	386 (29.5)	204 (22.5)	13.2 (1)	<.001
	Mental Health	538 (41.2)	306 (33.7)	12.2 (1)	<.001
	Communication with HCP	347 (26.5)	240 (26.5)	0.0 (1)	.99
	Health Behaviours	402 (30.8)	220 (24.3)	10.9 (1)	<.001
55	Mental Health				
	Reducing stress	261 (48.5)	165 (53.9)	2.1 (1)	.15
	Reducing anxiety and worry	266 (49.4)	148 (48.4)	0.0 (1)	.82
	Managing Low mood and depressive symptoms	250 (46.5)	135 (44.1)	0.3 (1)	.56
	Improving ability to control emotions	237 (44.1)	132 (43.1)	0.0 (1)	.85
	Being happier	240 (44.6)	154 (50.3)	2.3 (1)	.13
	Having better emotional well-being	260 (48.3)	162 (52.9)	1.5 (1)	.22
	Improving self-esteem	251 (46.7)	131 (42.8)	1.0 (1)	.31
	Improving confidence	252 (46.8)	133 (43.5)	0.8 (1)	.38
	Improving relationships with others	222 (41.2)	129 (42.2)	0.0 (1)	.86
57	Physical Health				
	Feeling less fatigued and tired	277 (71.8)	138 (67.6)	0.9 (1)	.34
	Having more energy	273 (70.7)	138 (67.6)	0.5 (1)	.50
	Sleeping better	267 (69.2)	139 (68.1)	0.0 (1)	.87
	Managing your weight	227 (58.8)	109 (53.4)	1.4 (1)	.24
	Improving your skin	229 (59.3)	117 (57.4)	0.1 (1)	.71
	Improving your fitness	237 (61.3)	114 (58.9)	1.5 (1)	.23
59	Health Behaviour				
	Increased activity/exercise	247 (61.4)	127 (57.7)	0.7 (1)	.41
	Improved diet	261 (64.9)	152 (69.1)	0.9 (1)	.34
	Better stress management	273 (67.9)	139 (63.2)	1.2 (1)	.27
	Reducing harmful habits	250 (62.2)	129 (58.6)	0.6 (1)	.43
	Better management of symptoms	314 (78.1)	162 (73.6)	1.3 (1)	.25
61	Sexual Health				
	STI's and how to avoid them	326 (61.0)	136 (44.7)	20.9 (1)	<.001
	The signs and symptoms of STI's	312 (58.4)	133 (43.7)	16.2 (1)	<.001
	Safe Sex	380 (71.2)	177 (58.2)	14.0 (1)	<.001
	Contraception Options	363 (68.0)	169 (55.6)	12.3 (1)	<.001
	The signs and symptoms during sex that are indicative of a health issue	376 (70.4)	183 (60.2)	8.7 (1)	.003
62	Improved Sex Life	385 (72.2)	223 (73.4)	0.1 (1)	.79
	More pleasure during sex	339 (63.6)	192 (63.2)	0.0 (1)	.96
	Better Understanding of your sexuality	374 (70.2)	198 (65.1)	2.0 (1)	.15
	Getting rid of sex related stigmas	347 (65.1)	193 (63.5)	0.2 (1)	.69
	Improved self love and self compassion	377 (70.7)	214 (70.4)	0.0 (1)	.98
64	Communication with HCP				
	Asking questions about my health/body	240 (80.4)	170 (71.7)	0.1 (1)	.8
	Asking for resources I think I need	167 (49.0)	107 (45.1)	0.7 (1)	.67
	Sharing with what's going on in my body	271 (79.4)	180 (75.9)	0.8 (1)	.37
	Understanding of my reproductive health	253 (74.2)	171 (72.2)	0.2 (1)	.65

[a]All general health questions were compared for participant responses from ‘a moderate to a great deal’.
